# Cytotoxic Sesterterpenes from Thai Marine Sponge *Hyrtios erectus*

**DOI:** 10.3390/md16120474

**Published:** 2018-11-28

**Authors:** Wirongrong Kaweetripob, Chulabhorn Mahidol, Pittaya Tuntiwachwuttikul, Somsak Ruchirawat, Hunsa Prawat

**Affiliations:** 1 Chulabhorn Research Institute, Kamphaeng Phet 6 Road, Bangkok 10210, Thailand; Kwirongrong@cri.or.th (W.K.); mahidol_natlab@cri.or.th (C.M.); Somsak@cri.or.th (S.R.); 2 Chulabhorn Graduate Institute, Chemical Biology Program, Chulabhorn Royal Academy, Kamphaeng Phet 6 Rood, Bangkok 10210, Thailand; 3 Laboratory of Natural Products Chemistry, Faculty of Science and Technology, Phuket Rajabhat University, Phuket 83000, Thailand; pittaya_tun@yahoo.co.th; 4 Center of Excellence on Environmental Health and Toxicology (EHT), CHE, Ministry of Education, Bangkok 10210, Thailand

**Keywords:** *Hyrtios erectus* (CRI 572 and CRI 588), Thorectidae, manoalides, scalarane, 2-furanone derivatives, cytotoxic activities

## Abstract

Four sesterterpenes, erectusolides B, C, D, and seco-manoalide-25-methyl ether, two 2-furanone derivatives, erectusfuranones A and B, together with thirteen known sesterterpenes, (6*Z*)-neomanoalide-24-acetate, two diastereomers of 24-*O*-methylmanoalide, luffariolide B, manoalide, (6*E*)- and (6*Z*)-neomanoalide, seco-manoalide, scalarafuran, 12-acetylscalarolide, 12-epi-*O*-deacetyl-19-deoxyscalarin, 12-epi-scalarin, and 12-*O*-deacetyl-12-epi-scalarin, three indole alkaloids, 5-hydroxy-1*H*-indole-3-carbaldehyde, hyrtiosine A, and variabine B, and one norterpene, cavernosine were isolated from the marine sponge *Hyrtios erectus*. Their structures were determined by means of spectroscopic methods and the absolute configurations of the asymmetric centers were determined using the modified Mosher’s method. The cytotoxic activities for the isolated compounds have been reported.

## 1. Introduction

**Figure 1:**
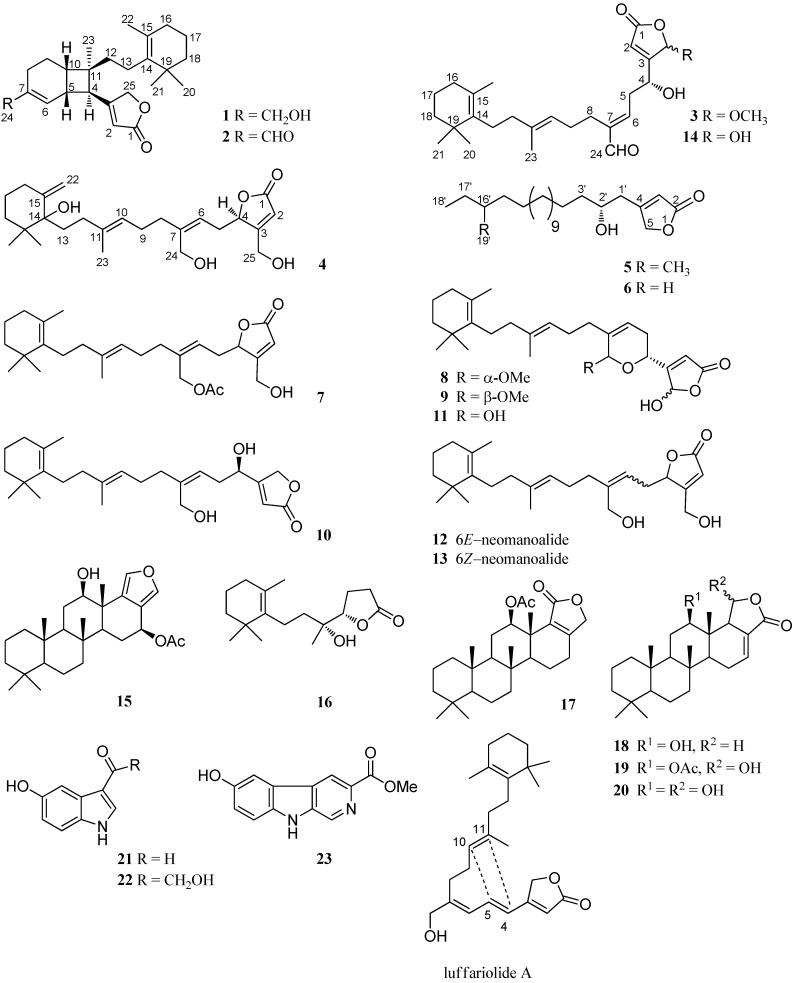
Isolated compounds **1**–**23** from sponge *Hyrtios erectus* and luffariolide A.

Marine organisms have always been an attractive source of natural products with novel and exotic structures and useful biological activities [1,2,3,4,5,6,7,8,9,10,11]. Marine sponges of the genus *Hyrtios* (order Dictyoceratida family Thorectidae) have yielded scalarane [6,8,12,13,14] and manoalide [12] types sesterterpenes, which are important groups of active secondary metabolites. They have been reported to possess many biological activities, such as cytotoxic [3,6,15,16], antibacterial [9,11], antibiotic [17] activities, inhibition of the DNA-relaxing activity of mouse DNA topoisomerase I [12], and enhancement nerve growth factor synthesis in cultured astroglial cells [13]. As a part of our ongoing research program focused on the discovery of cytotoxic compounds from Thai marine sponges [18,19,20], drawing from the previous report, one sesterterpene, erectusolide A, six phenolic alkenes, erectuseneols A–F, together with nine known compounds, were isolated from the EtOAc soluble extract of *Hyrtios erectus* (Chulabhorn Research Institute (CRI) 588) [3]. Of these, some sesterterpenes exhibited significant cytotoxic activities against the MOLT-3 cell line with an IC_50_ values of 3.79–5.82 μM [3]. Accordingly, further investigation of the EtOAc soluble extract was carried out, leading to the isolation of four new sesterterpenes, erectusolides B (**1**), C (**2**), D (**4**), and seco-manoalide-25-methyl ether (**3**), two new 2-furanone derivatives, erectusfuranones A and B (**5** and **6**), together with seventeen known sesterterpenes, which were identified as (6*Z*)-neomanoalide-24-acetate (**7**) [9], two diastereomers of 24-*O*-methylmanoalide (**8** and **9**) [10], luffariolide B (**10**) [15], manoalide (**11**) [10,12,17], (6*E*)- and (6*Z*)-neomanoalides (**12** and **13**) [12,15,17], seco-manoalide (**14**) [12,17] scalarafuran (**15**) [8,16], 12-acetylscalarolide (**17**) [21], 12-epi-*O*-deacetyl-19-deoxyscalarin (**18**) [8,16], 12-epi-scalarin (**19**) [13,16], and 12-*O*-deacetyl-12-epi-scalarin (**20**) [13,14,16], three indole alkaloids, 5-hydroxy-1*H*-indole- 3-carbaldehyde (**21**) [14,22], hyrtiosine A (**22**) [14,22], and variabine B (**23**) [7], and one norterpene, cavernosine (**16**) [3,23]. All of the known compounds (Figure 1) were readily identified by extensive study of their spectral data, including high resolution atmospheric pressure chemical ionization mass spectrometry (HRAPCIMS) or high resolution electrospray ionization mass spectrometry (HRESIMS), 1D and 2D nuclear magnetic resonance (NMR) data, as well as by comparison with those reported in the literatures.

## 2. Results

Two separate collections of *H. erectus* were studied; and they were collected in February 2011 from different locations of the Similan Island, Phangnga province, Thailand. The MeOH extract of the Thai marine sponge *H. erectus* from collection CRI 588 was dissolved in H_2_O and partitioned with EtOAc. The EtOAc soluble extract was fractionated by column chromatography over silica gel, Sephadex LH-20, and reversed-phase high performance liquid chromatography (HPLC) to afford three new sesterterpenes, erectusolides B (**1**), C (**2**), and seco-manoalide-25-methyl ether (**3**), two new 2-furanone derivatives, erectusfuranones A (**5**) and B (**6**) together with eight known sesterterpenes, **7**–**14**. One additional new sesterterpene, erectusolide D (**4**), and ten known compounds (**13**, **15**–**23**) were obtained from the *H. erectus* collection CRI 572. All new compounds showed considerable structural similarity with the co-occurring known sesterterpenes.

**Figure 2:**
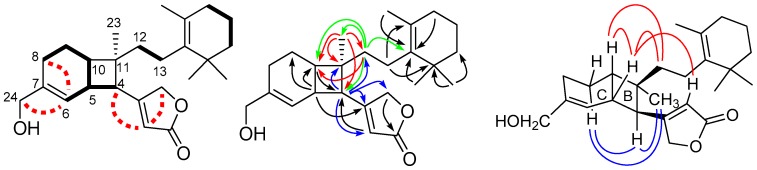
Key COSY (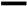 and 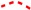), HMBC (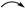), and NOESY (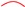) correlations of **1**.

Compound **1** was obtained as a weak optical rotation value (${\left[\alpha \right]}_{D}^{26}$ −1.3), and its molecular formula was determined to be C_25_H_36_O_3_ (eight degrees of unsaturation) by HRAPCIMS. Infrared (IR) absorption bands of compound **1** suggested the presence of *β*-substituted *α*,*β*-unsaturated *γ*-lactone (*β*-substituted butenolide) at 1779 and 1746 cm^−1^ and hydroxyl group at 3443 cm^−1^. Four of the eight degrees of unsaturation implied by the molecular formula of **1** were taken up in one carbon–oxygen double bonds and three carbon–carbon double bonds, thus indicating the tetracyclic nature of the molecule. The ^13^C and (distortionless enhancement by polarization transfer (DEPT) NMR spectra of **1** showed 25 carbons including, 4 tertiary methyls, 7 methylenes, 2 oxymethylenes, 3 methines, 2 olefinic methines, and 7 quaternary carbons. The ^1^H and ^13^C NMR spectral data of **1** (Table 1, Figures S1 and S2) were comparable to those of luffariolides A (Figure 1) and B (**10**) and implied that all compounds possessed identical two terminal units, which included a polyalkylated-cyclohexene (C_12_–C_22_) and *β*-substituted butenolide moieties. The major differences were found in the C_4_–C_11_, and C-23. In the ^1^H–^1^H COSY correlation of **1** (Figure 2), H-6 (δ_H_ 5.67) showed allylic coupling to H_ax_-8 (1H, δ_H_ 1.90–2.00) and H_2_-24 (2H, δ_H_ 4.03/4.06) and H-5 was coupled to H-4, H-6, and H-10. From its ^1^H–^1^H correlation spectroscopy (COSY) spectrum of **1** (Figure 2), typical allylic coupling of the olefinic methines at δ_H_ 5.86 (H-2) and 5.67 (H-6) with the respective methine signal at δ_H_ 2.74 (H-4) and oxymethylene at δ_H_ 4.03/4.06 (H_2_-24) were discernable. Both the COSY and heteronuclear multiple bond correlation (HMBC) data indicated that the double bonds were not conjugated to each other. The ^1^H, ^13^C, and COSY NMR data confirmed the presence of a 3-hydroxymethylbicyclo [4.2.0] oct-2-ene (C_4_–C_11_ and C-24) in the molecule. The HMBC correlations of **1** (Figure 2 and Figure S4), the proton signal at δ_H_ 5.86 (H-2) correlated to methine carbon at δ_C_ 51.0 (C-4), H-4 at δ_H_ 2.74 correlated to quaternary carbon at δ_C_ 169.7 (C-3), methine carbon at δ_C_ 115.3 (C-2), and methylene carbon at δ_C_ 73.2 (C-25) allowed the connectivity of the butenolide with the bicyclic ring through the linkage of C-3 and C-4. The linkage of the polyalkylated-cyclohexene and CH_3_-23 at quaternary C-11 on the molecule were deduced from the HMBC correlations of H_3_-23 (δ_H_ 1.13) with C-4, C-10 (δ 37.0), C-11 (δ 43.0), and C-12 (δ 36.6) and the correlations of H_2_-12 (δ_H_ 1.28–1.36 and 1.58–1.66) with C-4, C-10, C-14 (δ 136.3), and C-23 (δ 21.5). The large trans-diaxial coupling constant between H_ax_-9 and H_ax_-10 (*J* = 12.2 Hz) on the cyclohexene ring suggested the *β*-configuration of H_ax_-10. The relative stereochemistry of **1** was established by analysis of the (nuclear overhauser effect spectroscopy (NOESY) spectrum (in CDCl_3_ and C_6_D_6_; Figure 2). NOESY cross-peaks resonance of H-4, H_α_-9, and H_3_-23 and H-5, H-10, H-12, and H-2 implied a *cis*-junction for rings B/C and H-5, H_ax_-10, *β*-substituted butenolide moiety, and polyalkylated-cyclohexene (C_12_–C_22_) are on the same face while the methyl group (CH_3_-23) and H-4 on the opposite face of the molecule. Structure **1** with relative stereochemistry as shown was thus assigned to erectusolide B, which contained fused cyclohexene and cyclobutane rings. It could derive from luffariolide A (Figure 1) [11], through a biogenetic pathway involving a 2 + 2 cycloaddition of double bonds at C-4/C-5 and C-10/C-11 as suggested by Lin, H.W. and co-workers [4]. Furthermore, compounds **1** and **2** represent two important compounds which lend further support for the proposed biosynthetic pathway of Lin et. al. for the cyclobutane formation [4]. This substance has an optical rotation near zero (${\left[\alpha \right]}_{D}^{26}$ −1.3); attempting to separate the substance using various chiral columns was found to be inseparable.

Compound **2** was obtained as optically active (${\left[\alpha \right]}_{D}^{26}$ −18.5), and its molecular formula was determined to be C_25_H_34_O_3_ (nine degrees of unsaturation) by HRAPCIMS. Infrared (IR) absorption bands of compound **2** suggested the presence of *β*-substituted *α*,*β*-unsaturated *γ*-lactone at 1779 and 1746 cm^−1^ and *α*,*β*-unsaturated carbonyl group at 1682 cm^−1^. The ^1^H and ^13^C NMR spectroscopic data of **2** (Table 1, Figures S4–S8) indicated that it was essentially identical to compound **1**, except for the presence of an aldehyde group (δ_H_ 9.45, s; δ_C_ 192.9) for **2** in place of the hydroxylmethyl group (δ_H_ 4.03/4.06, each d, *J* = 13.2 Hz, δ_C_ 66.8) for **1**. This was further confirmed by the HMBC correlations (Figures S11 and S12) between olefinic H-6 (δ_H_ 6.74) and aldehydic carbon (δ_C_ 192.9) and between proton aldehyde (δ_H_ 9.45) and C-7 (δ_C_ 143.7) and C-8 (δ_C_ 19.4). The NOESY spectrum of **2** was similar to that observed of **1** indicating the same relative stereochemistry. Thus, compound **2** was suggested to be the formaldehyde analog of **1**, and named erectusolide C. This compound has low optical rotation (${\left[\alpha \right]}_{D}^{26}$ −18.5); attempting to separate the compound using various chiral columns was unsuccessful. 

**Figure 3:**
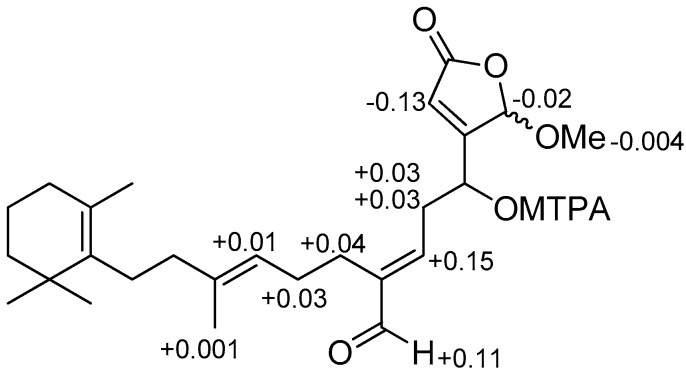
Δδ*_S_*_-*R*_ values in ppm for *S*- and *R*-MTPA esters of compound **3** in CDCl_3_.

Compound **3** was obtained as a pale yellow solid, exhibiting similar ultraviolet (UV), infrared (IR), ^1^H, and ^13^C NMR spectra (Table 2, Figures S13 and S14) as (6*E*) seco-manoalide (**14**) [17]. Accurate mass measurement by HRAPCIMS of **3** indicated a pseudo molecular ion peak at *m/z* 465.2414 [M + Cl]^−^ (calcd for C_26_H_38_ClO_5_, 465.2413), consistent with the molecular formula C_26_H_38_O_5_. This difference of 14 amu compared to the molecular formula of **14**, and the appearance of a signal of methoxy group in ^1^H, and ^13^C NMR spectra of **3** at δ_H_ 3.65 and δ_C_ 57.9. It is suggested that the hydroxyl group at C-25 was replaced by a methoxyl group. This was further confirmed by the HMBC correlations (Figure S16) between the methoxyl protons (δ_H_ 3.65) and hemiacetal carbon (δ_C_ 103.3) and between H-25 (δ_H_ 5.84) and methoxyl carbon (δ_C_ 57.9). The absolute stereochemistry at C-4 of **3** was determined by the modified Mosher’s method [24]. The hydroxyl group of **3** was converted into both the *S*- and *R*-MTPA esters **3a** and **3b**, respectively. The ^1^H NMR chemical shifts were assigned by the analysis of the ^1^H–^1^H COSY NMR data for each MTPA ester (experimental section). The calculated Δδ*_S_*_−*R*_ values were positive for the H_2_-5 (+0.03 and +0.03), H-6 (+0.15), H_2_-8 (+0.04), and H-24 (+0.11) and negative for the H-2 (−0.13), 25-OMe (−0.004), and H-25 (−0.02) (Figure 3), implying that the absolute configuration of C-4 was *R*. Thus, compound **3** was characterized as (4*R*,6*E*) seco-manoalide-25-methyl ether.

Compound **4** was obtained as an optically active (${\left[\alpha \right]}_{D}^{26}$ −20.9), and its molecular formula was determined to be C_25_H_38_O_5_ (seven degrees of unsaturation) by HRAPCIMS. Five of the seven degrees of unsaturation implied by the molecular formula of **4** were taken up in one carbon–oxygen double bonds and four carbon–carbon double bonds, thus indicating the bicyclic nature of the molecule. The IR spectrum exhibited absorption bands corresponding to a hydroxyl group (3424 cm^−1^), an ester carbonyl (1739 cm^−1^), and an exomethylene substituent (898 cm^−1^). Similarities in the NMR spectra between compounds **4** (Table 2, Figures S17 and S18) and 6*Z*-neomanoalide (**13**) suggested that compound **4** was also a neomanoalide-type sesterterpene [17].

**Figure 4:**
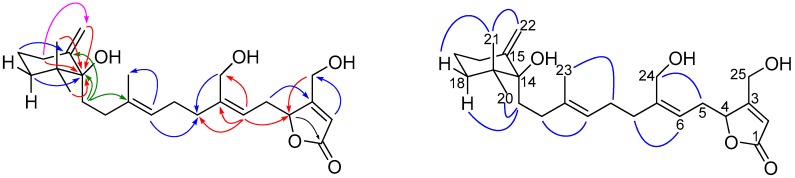
Key HMBC (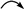), and NOESY (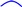) correlations of **4**.

The main differences in the ^1^H NMR spectra of **4** and compound **13** were the absence of one olefinic methyl group resonance in compound **4** and the appearance of a resonance attributable to an exomethylene moiety (δ_H_ 4.83 and 4.91, H_2_-22). Observation of a sp^2^ methylene carbon resonance (δ_C_ 108.3) and a corresponding quaternary carbon (δ_C_ 150.5) further supported the presence of a exocyclic methylene functionality in the structure of **4**. An oxygenated quaternary carbon (δ_C_ 80.4, C-14) was observed by ^13^C NMR and DEPT experiments. The assignment and placement of the hydroxyl substituent at C-14 and the exocyclic methylene at C-15 were deduced by observation of long range ^1^H–^13^C HMBC correlations (Figure 4 and Figure S20) (H_2_-13, H_2_-16, H_2_-18, H_3_-21/22 to C-14, H_2_-17 to C-15, and H_2_-16 to C-22). The relative stereochemistry of the methylenecyclohexane unit was elucidated mainly on the basis of NOESY correlations (Figure 4). The correlations among axial H_α_-18 (δ_H_ 1.63, td, *J* = 13.0, 5.7 Hz), axial methylene H_2_-13, and equatorial CH_3_-20 (δ_H_ 0.96) and correlations among axial H_3_-21 (δ_H_ 0.88), exocyclic methylene H_2_-22, and equatorial H_β_-18 (δ_H_ 1.37, brd, *J* = 13.7 Hz) that were observed in **4** indicated that the equatorial hydroxyl group at C-14 occupied the *β*-face. The geometry of the olifinic bonds was assigned as 6*Z*,10*E* on the basis of NOESY correlations between H_2_-5 and H_2_-24 and between H_2_-9 and H_3_-23, respectively. Compound **4** exhibited a negative optical rotation {${\left[\alpha \right]}_{D}^{25}$ −20.9 (*c* 1.64, CHCl_3_)}, similar to that of 6*Z*-neomanoalide (**13**) {${\left[\alpha \right]}_{D}^{25}$ −28 (*c* 0.8, CH_2_Cl_2_)} [12] the relative stereochemistry at C-4 of **4** was then assigned to be *R*. Thus, the structure **4** was concluded as shown in Figure 1 and was named erectusolide D.

**Figure 5:**
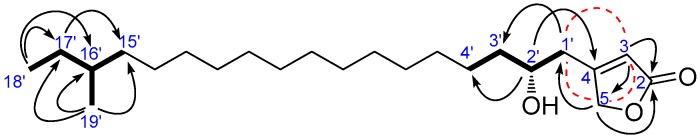
Key COSY (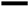), long rang COSY (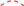), and HMBC (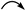) correlations of **5**.

**Figure 6:**
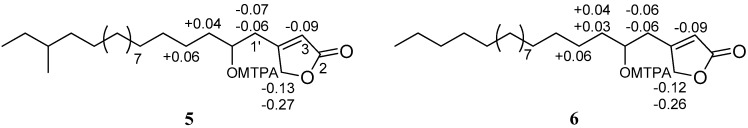
Δδ*_S_*_-*R*_ values in ppm for *S*- and *R*-MTPA esters of compounds **5** and **6** in CDCl_3_.

Compound **5** was obtained as optically active (${\left[\alpha \right]}_{D}^{25}$ +1.3), and its molecular formula was determined to be C_23_H_42_O_3_ (three degrees of unsaturation) by HRESIMS. The IR absorption bands data of compound **5** suggested the presence of *β*-substituted *α*, *β*-unsaturated *γ*-lactone (*β*-substituted butenolide for three degrees of unsaturation in the structure at 1777 and 1739 cm^−1^ in addition to the hydroxyl group at 3442 cm^−1^. The ^1^H and ^13^C NMR spectra of **5** as shown in Table 3 (Figures S11 and S22) suggests that compound **5** possess *β*-substituted butenolide moiety to which a saturated long chain hydrocarbon group is attached. The ^1^H NMR signals at δ_H_ 5.93 (1H) and 4.85 (2H) assignable to an olefinic proton and methylene protons at the *α*- and *γ*-positions of the *α*,*β*-unsaturated *γ*-lactone, respectively, reveals that the long chain hydrocarbon group is attached at the *β*-position (C-4). The 2-hydroxy-16-methyloctadecane group (long chain hydrocarbon) was assigned by a combination of HRESIMS, ^1^H and ^13^C NMR, DEPT, COSY, HSQC (Figure S23), and HMBC analyses. The HMBC spectrum of **5** (Figure 5 and Figure S24) showed the correlations from two of the methyl protons at δ_H_ 0.85 (H_3_-18′, t, *J* = 7.2 Hz) and δ_H_ 0.84 (H_3_-19′, d, *J* = 6.0 Hz) to the methine carbon at δ_C_ 34.4 (C-16′) and methylene carbon at δ_C_ 29.42 (C-17′) suggesting that the position of the second methyl group was attached at C-16′. In the HMBC spectrum of **5** also showed the correlations between methylene H_2_-1′ (δ_H_ 2.52/2.64) and C-3 (δ_C_ 117.3), C-2′ (δ_C_ 70.2), C-3′ (δ_C_ 37.7), and C-5 (δ_C_ 73.8) and between H-2′ (δ_H_ 3.84–3.90) and C-4 (δ_C_ 167.3), C-4′ (δ_C_ 25.5) indicating clearly that the methylene carbon (C-1′) of 2-hydroxy-16-methyloctadecyl attached to the C-4 of the *α*,*β*-unsaturated *γ*-lactone. The absolute stereochemistry at C-2′ of **5** was determined by the modified Mosher’s method [24]. The hydroxyl group of **5** was converted into both the *S*- and *R*-MTPA esters **5a** and **5b**, respectively, each of which was a single diastereoisomer by ^1^H and COSY NMR experiments. The calculated Δδ*_S_*_-*R*_ values were positive for the H_2_-3′ (+0.04) and H_2_-4′ (+0.06), while Δδ*_S_*_-*R*_ values were negative for H_2_-1′ (−0.07 and −0.06), H-3 (−0.09), and H_2_-5 (−0.13 and −0.27) (Figure 6), which implied the absolute configuration of C-2′ was *R*. Thus, compound **5** was characterized as 4-((2*R*)-2-hydroxy-16-methyloctadecyl)furan-2(5*H*)-one and was named erectusfuranone A.

Compound **6** was obtained as optically active (${\left[\alpha \right]}_{D}^{26}$ −4.9), and its molecular formula was determined to be C_22_H_40_O_3_ (three degrees of unsaturation) by HRESIMS. The UV, IR, ^1^H and ^13^C NMR (Figures S25 and S26) spectroscopic data of **6** indicated that it was essentially identical to compound **5**. The signals of a methine moiety (C-16′; δ_C_ 34.3, δ_H_ 1.23–1.37) and a methyl group (C-19′; δ_C_ 19.2, δ_H_ 0.84) were not evident in **6**, instead a methylene group (C-16′; δ_C_ 31.9, δ_H_ 1.22–1.31) was observed. The absolute stereochemistry at C-2′ of **6** was determined by the modified Mosher’s method [24]. The hydroxyl group of **6** was converted into both the *S*- and *R*-MTPA esters **6a** and **6b**, respectively, each of which was a single diastereoisomer judged by ^1^H and COSY NMR experiments. The Δδ*_S_*_-*R*_ (Figure 6) values observed in the ^1^H NMR spectra were calculated and the resulting positive Δδ values for H_2_-6 (+0.04 and +0.04) and H_2_-7 (+0.06), and negative Δδ values for H_2_-1′ (−0.06 and −0.06), H-3 (−0.09), and H_2_-5 (−0.12 and −0.26) were consistent with the 2′*R* configuration. Thus, compound **6** was characterized as (*R*)-4-(2-hydroxyoctadecyl) furan-2(5*H*)-one and was named erectusfuranone B.

Cytotoxicity of the isolated compounds **3**, **5**, **7**–**20** and **23** were evaluated against several cancer cell lines such as (Table 4), MOLT-3 (acute lymphoblastic leukemia), HepG2 (hepatocarcinoma), HeLa (human cervical carcinoma), HuCCA-1 (human chlolangiocarcinoma), A549 (non-small-cell lung cancer), H69AR (multidrug resistance small-cell lung cancer), KB (human epidermoid carcinoma in the mouth), T47D (hormone dependent breast cancer), MDA-MB-231 (hormone independent breast cancer), and MRC-5 (normal embryonic lung cell). Compounds **8**, **9**, **14** and **20** showed good cytotoxic activity against MOLT-3 cell line with IC_50_ values of 1.77, 1.30, 6.68, and 7.64 μM, respectively, compounds **8** and **9** also showed cytotoxic activity against HeLa, HuCCA-1, and A549 cell lines with IC_50_ values of 1.51–16.77 μM. In addition, compounds **8** and **20** selectively exhibited cytotoxic activity toward the MOLT-3 (IC_50_ 1.77 and 7.64 µM, respectively) cancer cell line with the selectivity index (SI) value of 7 (IC_50_ 13.00 µM for normal cell line, MRC-5) and 9 (IC_50_ 70.97 µM for MRC-5), respectively. The SI value is the ratio of IC_50_ of normal cell (MRC-5) and IC_50_ of cancer cell line. Compound **9** selectively exhibited cytotoxic activity against MOLT-3 (IC_50_ 1.30 µM) and HeLa (IC_50_ 1.51 µM) cell lines with respective SI values of 8 and 7 (IC_50_ 10.84 µM for MRC-5). Manoalides **8** and **9**, the acetal derivatives of the hemiacetal **11**, showed higher activity than **11**, suggesting that the presence of 24-*O*-methyl of manoalides **8** and **9** were important for cytotoxic activity (Table 4). Manoalide **11** was previously reported to possess good cytotoxicity against L1210 (mouse lymphocytic leukemia) and KB (mouth epidermal carcinoma) cell lines with the IC_50_ value of 0.053 and 0.63 µM, respectively [12]. Compound **20** (12-*O*-deacetyl-12-epi-scalarin) exhibited cytotoxic activity against A549 cells with an IC_50_ of 73.38 µM (Table 4), while its IC_50_ have been reported in the literature to be 36.82 μM [16]. Luffariolide B (**10**), (6*E*)- and (6*Z*)-neomanoalides (**12** and **13**) showed weak cytotoxicity against the MOLT-3 cell line with IC_50_ of 35.45, 37.81, and 34.10 μM, respectively, but they exhibited cytotoxicity against the L1210 cell line with IC_50_ of 3.23, 24.38, and 13.93 μM, respectively [15].

## 3. Experimental Section

### 3.1. General Experimental Procedures 

Optical rotations were recorded on a JASCO DIP 1020 polarimeter using cylindrical glass cell (10 mm inner diameter (I.D.) × 10 mm). UV spectra were measured with a UV-1700 Pharma Spec (Shimadzu, Kyoto, Japan) spectrophotometer. Fourier transform infrared (FTIR) spectra were obtained using a universal attenuated total reflectance attached on Perkin Elmer Spectrum One spectrometer (PerkinElmer, Waltham, MA, USA). Nuclear magnetic resonance (NMR) spectra were recorded in a CDCl_3_ or C_6_D_6_ solution containing Me_4_Si as internal standard on Bruker AM400 or AVANCE600 spectrometer (Bruker Corporation, Billerica, MA, USA). HR–MS was performed on a Bruker (Micro ToF, Bruker Corporation, Billerica, MA, USA) spectrometer. HPLC was carried out on a Waters 600 system (Warers Corporation, Milford, MA, USA) equipped with a Waters Delta 600 pump, a Waters 600 Controller, a Waters 2998 photodiode array detector, and Waters Empower 2 software. Sephadex^™^ LH-20 (GE Healthcare Bio-Sciences AB, Uppsala, Sweden) was used for a column gel filtration. All commercial grade solvents were distilled prior to use and spectral grade solvents were used for spectroscopic measurements.

### 3.2. Sponge Material

Sponges (*Hyrtios erectus*) CRI 572 and CRI 588 were collected by hand using scuba at a depth of 30–40 feet in the Similan Island at the Andaman Sea (Phangnga province, Thailand) on 22 and 23 February 2011, respectively. The sponges were identified by Dr. Sumaitt Putchakarn, Head of Marine Biodiversity Research, Unit Curator of Porifera and Echinodermata, Institute of Marine Science, Burapha University, Bangsaen, Chonburi, Thailand. The voucher specimens (CRI 572 and CRI 588) were presently deposited at the Laboratory of Natural Products, Chulabhorn Research Institute, Bangkok, Thailand. 

### 3.3. Extraction and Isolation

A frozen sample (2.6 kg) of *H. erectus* collection CRI 572 was cut into small pieces and extracted exhaustively with MeOH. The extract was filtered through cotton, and then evaporated under reduced pressure to give an aqueous residue, which was partitioned with EtOAc. The organic layer was concentrated to give a dark brown solid (7.52 g). The EtOAc–soluble fraction was subjected to vacuum liquid chromatography on silica gel and eluted with an EtOAc–hexane gradient (0→100% of EtOAc). Nine fractions (F1–F9) were obtained. F2 (340.5 mg) was subjected to column chromatography on Sephadex LH-20, using CH_2_Cl_2_–MeOH (1:1) to give compound **15** (16 mg). F4 (293.3 mg) was repeatedly fractionated by Sephadex LH-20, using CH_2_Cl_2_–MeOH (1:1) to give a mixture compounds (96.8 mg) that was separated using on a HPLC column (Hichrome C18, 5 µm, 21.2 mm × 250 mm with MeOH–H_2_O (gradient, 77→97% MeOH for 40 min), flowrate 12 mL/min, λ 220, 265 nm) to afford compounds **16** (28 mg, at 12.7 min), **17** (4.9 mg, at 32.5 min). F5 (574.4 mg) was subjected to column chromatography on Sephadex LH-20, using 100% MeOH to obtain a mixtures compound (9.3 mg) that was separated using on a HPLC column (Sunfire C18, 5 µm, 10 mm × 250 mm, with 80% CH_3_CN–H_2_O, flowrate 3 mL/min, λ 220 nm) to afford **18** (4.0 mg, at 25.7 min). F6 (976.4 mg) was subjected to column chromatography on Sephadex LH-20, using CH_2_Cl_2_–MeOH (1:1) to give three fractions (f1–f3). Fraction f1 was subjected to repeated chromatography on Sephadex LH-20, using 100% MeOH to give a mixtures compound (73.5 mg) that was separated using a HPLC column (Hichrome C18, 5 µm, 21.2 mm × 250 mm, with MeOH–H_2_O (gradient, 75→100% MeOH for 50 min), flowrate 12 mL/min, λ 220 nm) to afford compound **19** (2.3 mg, at 35.2 min). Fraction f2 was subjected to column chromatography on Sephadex LH-20, using 100% MeOH to give a mixtures compound (45.6 mg) that was separated using on a HPLC column (Sunfire C18, 5 µm, 19 mm × 250 mm, with 88% MeOH–H_2_O, flowrate 12 mL/min, λ 220 nm) to afford **20** (20 mg, at 16.7 min). Fraction f3 was subjected to repeated chromatography on Sephadex LH-20, using 100% MeOH to give a mixtures compound (6.9 mg) that was separated using on a preparative thin layer chromatography (PTLC) [hexane–CH_2_Cl_2_–acetone (2:2:1) as eluent] to afford **21** (1.4 mg). F7 (508.1 mg) was subjected to column chromatography on Sephadex LH-20, using 100% MeOH to give a mixtures compound (12.1 mg) that was separated using on a HPLC column (Sunfire C18, 5 µm, 19 mm × 250 mm, with 20% MeOH–H_2_O, flowrate 12 mL/min, λ 254 nm) to afford compound **22** (7.5 mg, at 12.8 min). F8 (298.1 mg) was repeatedly fractionated by Sephadex LH-20, using CH_2_Cl_2_–MeOH (1:1) to give a mixture compound (127.6 mg) that was separated using a HPLC column (Hichrome C18, 5 µm, 21.2 mm × 250 mm with 75% MeOH–H_2_O, flowrate 12 mL/min, λ 222 nm) to afford compounds **4** (16.4 mg, at 11.3 min), and **13** (16.1 mg, at 33.5 min). F9 (249.2 mg) was subjected to repeated chromatography on Sephadex LH-20, using CH_2_Cl_2_–MeOH (1:1) to give a mixtures compound (20.3 mg) that was separated using a HPLC column (Hichrome C18, 5 µm, 21.2 mm × 250 mm, with MeOH–H_2_O (45–55% MeOH for 20 min), flowrate 12 mL/min, λ 234 nm) to afford compound **23** (5.4 mg, at 14.7 min). The flesh sponge *Hyrtios erectus* collection CRI 588 (14.0 kg) was cut into small pieces and extracted repeatedly with MeOH (20 L) three times (3 × 20 L). After the evaporation of the solvent, the concentrated MeOH extracts was partitioned between EtOAc and water and the EtOAc fraction was chromatographed on a silica gel column with CH_2_Cl_2_–hexane (1:1) containing increasing proportions of acetone as eluent, to give 7 fractions (A–G). Fraction C (4.0 g) was fractionated by Sephadex LH-20 with MeOH–CH_2_Cl_2_ (1:1) as eluent, to give 2 fractions (C1–C2). C2 (230.3 mg) was repeatedly chromatography on Sephadex LH-20, using CH_2_Cl_2_–MeOH (4:1) to give a mixtures compound (200 mg) that was separated using a HPLC column (Sunfire Prep C18, 5 µm, 10 mm × 250 mm) with MeOH–H_2_O (93:7), flowrate 2.5 mL/min, λ 224 nm, to afford compounds **5** (4.1 mg at 45.5 min), and **6** (7.2 mg at 48.2 min). Fraction D (9.0 g) was further fractionated on a Sephadex LH-20 column chromatography with MeOH–CH_2_Cl_2_ (1:1) to yield 4 fractions (D1–D4). D4 (400 mg) was further purified by HPLC column (Sunfire C18, 5 µm, 19 mm × 250 mm) with CH_3_CN–H_2_O (80:20), flowrate 8 mL/min, λ 224, 236 nm, to afford compounds **1** (5.9 mg, at 23.0 min), **2** (1.6 mg, at 29.6 min), and **3** (7.2 mg, at 35.6 min). Fraction E (25.0 g) was further fractionated by vacuum liquid chromatography eluted with CH_2_Cl_2_–hexane (1:1) containing increasing proportions of acetone as eluent, to give 4 fractions (E1–E4). Fraction E1 (2 g) was further purified by a HPLC column (Sunfire C18, 5 µm, 19 mm × 250 mm) with CH_3_CN–H_2_O (85:15), flowrate 8 mL/min, λ 224, 236 nm, to afford compounds **7** (74 mg, at 32.3 min), **8** (52 mg, at 47.9 min), and **9** (229 mg, 52.8 min). Fraction E3 (3 g) was further purified by a HPLC column (Sunfire C18, 5 µm, 19 mm × 250 mm) with CH_3_CN–H_2_O (80:20), flowrate 8 mL/min, λ 224, 236 nm, to afford compounds **10** (27 mg, at 19.9 min), and **11** (56 mg, at 32.5 min). Fraction E4 (1.4 g) was further purified by HPLC column (Sunfire C18, 5 µm, 19 mm × 250 mm) with CH_3_CN–H_2_O (82:18), flowrate 8 mL/min, λ 224, 236 nm, to afford compounds **13** (72 mg, at 27.35 min) and **14** (33 mg, at 29.3 min). Fraction G (4.0 g) was further filtrated on a Sephadex LH-20 column chromatography with MeOH to give a residue (655 mg), which was further purified by HPLC column (Cosmosil, 5 μ C18-MS-II, 20 mm × 250 mm) with CH_3_CN–H_2_O (60:40), flowrate 8 mL/min, λ 224, 236 nm, to afford compound **12** (70.0 mg, at 64.2 min).

Preparation of (*S*)- and (*R*)-MTPA Esters of compound **3**: To a solution of **3** (3.1 mg) in pyridine (0.9 mL) was added (*R*)-MTPA-chloride (17 µL). The mixture was stirred at room temperature for 3 h, checked with thin layer chromatography (TLC) to make sure that the reaction was complete, quenched by the addition of 2 mL of H_2_O, and the mixture was subsequently extracted with CH_2_Cl_2_ (2 mL) three times (3 × 2 mL). The CH_2_Cl_2_ soluble layers were combined, dried over anhydrous MgSO_4_, and evaporated. The residue was subjected to short silica gel column chromatography using hexane–EtOAc (4:1) to give the (*S*)-MTPA ester **3a** (1.6 mg).

Selected ^1^H NMR (CDCl_3_ 600 MHz) of **3a**: δ_H_ 9.32, s, H-24; 6.28, t (*J* = 7.1 Hz), H-6; 5.86, brs, H-2; 5.79, s, H-25; 5.74, dd (*J* = 7.2, 4.2 Hz), H-4; 5.09, t (*J* = 7.1 Hz), H-10; 3.03, ddd (*J* = 16.1, 6.6, 4.2 Hz), Ha-5; 2.90, dt (*J* = 16.1, 8.0 Hz), Hb-5; 2.24, t (*J* = 7.6 Hz), H_2_-8; 2.02, m, H_2_-9. 

The same procedure was used to prepare the (*R*)-MTPA ester **3b** (2.5 mg from 3.9 mg of **3**) with (*S*)-MTPA chloride.

Selected ^1^H NMR (CDCl_3_ 600 MHz) of **3b**: δ_H_ 9.21, s, H-24; 6.13, t (*J* = 7.1 Hz), H-6; 6.00, brs, H-2; 5.81, s, H-25; 5.74, dd (*J* = 7.0, 4.2 Hz), H-4; 5.07, t (*J* = 6.9 Hz), H-10; 3.00, ddd (*J* = 16.2, 6.8, 4.3 Hz), Ha-5; 2.87, dt (*J* = 16.2, 7.5 Hz), Hb-5; 2.20, t (*J* = 7.7 Hz), H_2_-8; 1.99, m, H_2_-9

(*S*)-MTPA esters **5a** (1.3 mg from 1.4 mg of **5**) and **6a** (1.8 mg from 1.4 mg of **6**) and (*R*)-MTPA esters **5b** (1.5 mg from 1.4 mg of **5**), **6b** (1.7 mg from 1.2 mg of **6**) were prepared by the method mentioned above. 

Selected ^1^H NMR (CDCl_3_, 400 MHz) of **5a**: δ_H_ 5.77, brs, H-3; 2.72, dd (*J* = 15.7, 6.9 Hz), Ha-1′; 2.62, dd (*J* = 15.7, 3.1 Hz), Hb-1′; 5.28, m, H-2′; 1.67, m, H_2_-3′; 4.56, dd (*J* = 17.8, 1.8 Hz), Ha-5; 4.25, brd (*J* = 17.6 Hz), Hb-5. 

Selected ^1^H NMR (CDCl_3_, 400 MHz) of **5b**: δ_H_ 5.86, s, H-3; 2.78, dd (*J* = 15.7, 7.3 Hz), Ha-1′; 2.69, dd (*J* = 15.7, 3.1 Hz), Hb-1′; 5.29, m, H-2′; 1.63, m, H_2_-3′; 4.69, brd (*J* = 17.4 Hz), Ha-5; 4.52, brd (*J* = 17.4 Hz), Hb-5.

Selected ^1^H NMR (CDCl_3_, 600 MHz) of **6a**: δ_H_ 5.77, brs, H-3; 2.72, dd (*J* = 15.6, 7.1 Hz), Ha-1′; 2.63, dd (*J* = 15.6, 3.4 Hz), Hb-1′; 5.28, m, H-2′; 1.71, m, Ha-3′; 1.59, m, Hb-3′; 1.29, m, H_2_-4′; 4.57, dd (*J* = 17.8, 1.6 Hz), Ha-5; 4.26, brd (*J* = 16.7 Hz), Hb-5.

Selected ^1^H NMR (CDCl_3_, 600 MHz) of **6b**: δ_H_ 5.86, brs, H-2; 2.78, dd (*J* = 15.4, 6.58 Hz), Ha-1′; 2.69, dd (*J* = 15.4, 3.6 Hz), Hb-1′; 5.29, m, H-2′; 1.67, m, Ha-3′; 1.55, m, Hb-3′; 1.23, m, H_2_-4′; 4.69, d (*J* = 17.5 Hz), Ha-5; 4.52, d (*J* = 17.5 Hz), Hb-5.

Erectusolide B (**1**): Colorless powder; ${\left[\alpha \right]}_{D}^{26}$ −1.3 (*c* 0.56, CHCl_3_); UV λ_max_ (MeOH) nm (log *ε*) 202 (3.9), 224 (3.4), IR (ATR) *ν*_max_ 3443 (br), 2927, 2861, 1779, 1746, 1629, 1456, 1379, 1266, 1143, 1041, 888, 851, 735, 702 cm^−1^; ^1^H and ^13^C NMR data see Table 1; HRAPCIMS *m/z* 385.2727 [M + H]^+^ (calcd for C_25_H_37_O_3_, 385.2737).

Erectusolide C (**2**): Colorless powder; ${\left[\alpha \right]}_{D}^{26}$ −18.5 (*c* 0.16, CHCl_3_); UV λ_max_ (MeOH) nm (log *ε*) 234 (4.3); IR (ATR) *ν*_max_ 2924, 2855, 1779, 1748, 1682, 1632, 1458, 1378, 1261, 1142, 1040, 887 cm^−1^; ^1^H and ^13^C NMR data see Table 1; HRESIMS *m/z* 405.2390 [M + Na]^+^ (calcd for C_25_H_34_NaO_3_, 405.2400).

seco-Manoalide-25-methyl ether (**3**): Pale yellow solid; ${\left[\alpha \right]}_{D}^{24}$ −22.2 (*c* 1.86, CHCl_3_); UV λ_max_ (MeOH) nm (log *ε*) 203.5 (4.3), 232 (sh); IR (ATR) *ν*_max_ 3446 (br), 2930, 2863, 1762, 1687, 1457, 1371, 1203, 1120, 1070, 960, 898, 870, 735, 702 cm^−1^; ^1^H and ^13^C NMR data see Table 2; HRAPCIMS *m/z* 465.2414 [M + Cl]^−^ (calcd for C_26_H_38_ClO_5_, 465.2413).

Erectusolide D (**4**): Pale yellow gum; ${\left[\alpha \right]}_{D}^{25}$ −20.9 (*c* 1.64, CHCl_3_); UV λ_max_ (MeOH) nm (log *ε*) 202 (4.3), IR (ATR) *ν*_max_ 3424 (br), 2928, 2857, 1739, 1642, 1456, 1382, 1258, 1172, 1063, 898 cm^−1^; ^1^H and ^13^C NMR data see Table 2; HRAPCIMS *m/z* 453.2418 [M + Cl]^−^ (calcd for C_25_H_38_ClO_5_, 453.2413).

erectusfuranone A (**5**): Colorless powder; ${\left[\alpha \right]}_{D}^{25}$ +1.3 (*c* 0.32, CHCl_3_); UV λ_max_ (MeOH) nm (log *ε*) 206 (3.9); IR (ATR) *ν*_max_ 3442 (br), 2923, 2853, 1777, 1739, 1637, 1457, 1378, 1175, 1027, 888, 720 cm^−1^; ^1^H and ^13^C NMR data see Table 3; HRAPCIMS *m/z* 367.3213 [M + H]^+^ (calcd for C_23_H_43_O_3_, 367.3207).

erectusfuranone B (**6**): Colorless powder; ${\left[\alpha \right]}_{D}^{26}$ −4.9 (*c* 0.61, CHCl_3_); UV λ_max_ (MeOH) nm (log *ε*) 206 (3.9); IR (ATR) *ν*_max_ 3402 (br), 2916, 2851, 1779, 1756, 1736, 1634, 1469, 1025, 892, 720 cm^−1^; ^1^H and ^13^C NMR data see Table 3; HRESIMS *m/z* 353.3053 [M + H]^+^ (calcd for C_22_H_41_O_3_, 353.3050).

### 3.4. Cytotoxicity Assay

Cytotoxic activity for adhesive cell lines including HepG2, HeLa, HuCCA-1, A549, H69AR, KB, T47D, MDA-MB-231, and MRC-5 cell lines were evaluated with the 3-(4,5-dimethylthiazol-2-yl)-2,5-diphenyltetrazoliumbromide (MTT) assay [25,26]. For the non-adhesive MOLT-3 cell line, the cytotoxicity was assessed using the 2,3-bis-(2-methoxy-4-nitro-5-sulphenyl-(2H)-tetrazolium-5-carboxanilide (XTT) assay [27]. Etoposide and doxorubicin were used as positive controls (Table 4)

## 4. Conclusions

Two separate collections of *H. erectus* were studied. Three new sesterterpenes, erectusolides B (**1**), C (**2**), and seco-manoalide-25-methyl ether (**3**), two new 2-furanone derivatives, erectusfuranones A (**5**) and B (**6**) together with eight known sesterterpenes, **7**–**14** were isolated from *H. erectus* collection CRI 588. One additional new sesterterpene, erectusolide D (**4**), and ten known compounds (**13**, **15**–**23**) were also obtained from the *H. erectus* collection CRI 572. Three sesterterpenes **8**, **9**, and **20** showed good cytotoxic activity against the MOLT-3 cell line with IC_50_ values of 1.30–7.64 μM and with SI (selectivity index) values 7–9.

## Figures and Tables

**Table 1:** ^1^H (600 MHz) and ^13^C (150 MHz) NMR data (^a^ in CDCl_3_^b^ in C_6_D_6_) of compounds **1** and **2**.

Position	1			2		
	^a^ δ_C_ (Type)	^a^ δ_H_, Mult. (*J* in Hz)	^b^ δ_H_, Mult. (*J* in Hz)	^a^ δ_C_ (Type)	^a^ δ_H_, Mult. (*J* in Hz)	^b^ δ_H_, Mult. (*J* in Hz)
1	174.0 (C)	-	-	173.3 (C)	-	-
2	115.3 (CH)	5.86, brd (1.5)	5.66, q (1.6)	116.0 (CH)	5.93, brd (1.6)	5.46, brs
3	169.7 (C)	-	-	168.1 (C)	-	-
4	51.0 (CH)	2.74, d (10.0)	2.24, d (10.1)	50.8 (CH)	2.85, d (9.9)	1.93, d (10.5)
5	33.3 (CH)	2.85, brt (9.0)	2.60–2.66, m	34.5 (CH)	3.14, brt (8.8)	2.40, brt (10.5)
6	122.1 (CH)	5.67, brd (0.9)	5.48, brd (1.4)	149.1 (CH)	6.74, t (3.0)	5.85, brt (3.1)
7	140.9 (C)	-	-	143.7 (C)	-	-
8	24.0 (CH_2_)	1.90–2.00, o 2.09, dt (16.4, 3.4)	1.76, brt (13.0) 1.90–1.98, o	19.4 (CH_2_)	1.86–1.93, o 2.65, dt (16.8, 3.6)	1.51–1.62, o 2.60, dt (13.3, 3.1)
9	22.8 (CH_2_)	1.65, qd (12.2, 4.2) 1.82–2.00, o	1.42, qd (12.3, 4.1) 1.57–1.67, m	22.3 (CH_2_)	1.50–1.58, o 1.88–1.99, o	0.88–0.96, m 1.27–1.37, 0
10	37.0 (CH)	2.25, dt (12.2, 6.9)	2.12, ddd (12.8, 7.9, 6.8)	38.0 (CH)	2.37, dt (11.7, 6.7)	1.82–1.88, o
11	43.0 (C)	-	-	43.7 (C)	-	-
12	36.6 (CH_2_)	1.08–1.20, m 1.49–1.61, o	1.28–1.36, m 1.58–1.66, o	36.6 (CH_2_)	1.15–1.18, o 1.55–1.58, o	1.10–1.17, m 1.35–1.40, o
13	22.4 (CH_2_)	1.90–2.00, o	1.94–2.06, o	22.2 (CH_2_)	1.88–1.99, o	1.23–1.40, o 1.78–1.84, o
14	136.3 (C)	-	-	136.0 (C)	-	-
15	127.5 (C)	-	-	127.7 (C)	-	-
16	32.7 (CH_2_)	1.89–1.98, o	1.92–2.06, o	32.7 (CH_2_)	1.85–1.99, o	1.78–1.89, o
17	19.4 (CH_2_)	1.53–1.62, o	1.60–1.68, o	19.4 (CH_2_)	1.52–1.62, o	1.49–1.58, o
18	39.7 (CH_2_)	1.40–1.44, m	1.49–1.52, m	39.7 (CH_2_)	1.40–1.44, m	1.37–1.42, o
19	35.1 (C)	-	-	35.1 (C)	-	-
20	28.71 (CH_3_)	0.96 *, s	1.14, s	28.74 (CH_3_)	0.96, s	0.99, s
21	28.72 (CH_3_)	0.97 *, s	1.11, s	28.7 (CH_3_)	0.98, s	1.02, s
22	19.8 (CH_3_)	1.55, s	1.66, s	19.8 (CH_3_)	1.56, s	1.52, s
23	21.5 (CH_3_)	1.13, s	0.88, s	21.5 (CH_3_)	1.16, s	0.63, s
24	66.8 (CH_2_)	4.03, d (13.2) 4.06, d (13.2)	3.88, s	192.9 (CH)	9.45, s	9.37, s
25	73.2 (CH_2_)	4.62, dd (17.4, 1.6) 4.65, dd (17.4, 1.0)	4.02, dd (17.1, 0.8) 4.16, dd (17.1, 1.7)	72.9 (CH_2_)	4.64, dd (17.3, 1.8) 4.68, dd (17.3, 1.1)	3.85, dd (17.2) 4.00, dd (17.2)

* Interchangeable; o = overlapped signals; Assignments are based on COSY, HSQC (Figures S3, S9 and S10), and HMBC experiments.

**Table 2:** ^1^H (600 MHz) and ^13^C (150 MHz) NMR data (CDCl_3_) of compounds **3** and **4**.

Position	3		4	
	δ_C_ (Type)	δ_H_, Mult. (*J* in Hz)	δ_C_ (Type)	δ_H_, Mult. (*J* in Hz)
1	169.2 (C)	-	172.7 (C)	-
2	119.3 (CH)	6.09, brt (1.3)	116.0 (CH)	6.04, brd (1.4)
3	166.3 (C)	-	171.7 (C)	-
4	66.9 (CH)	4.73, brdd (6.7, 4.1)	81.8 (CH)	5.08, m
5	34.7 (CH_2_)	2.78, dt (15.7, 7.5) 2.92, ddd (15.7, 7.1, 4.1)	30.2 (CH_2_)	2.57, ddd (15.0, 7.3, 5.5) 2.78, dt (15.0, 7.0)
6	147.0 (CH)	6.54, t (7.1)	119.9 (CH)	5.19, t (7.5)
7	146.1 (C)	-	143.0 (C)	-
8	24.6 (CH_2_)	2.32, t (7.4)	35.4 (CH_2_)	2.10–2.22, m
9	26.7 (CH_2_)	2.09, q (7.4)	26.3 (CH_2_)	2.07–2.17, m
10	122.4 (CH)	5.13, td (7.3, 1.0)	123.6 (CH)	5.07, m
11	137.5 (C)	-	136.6 (C)	
12	40.2 (CH_2_)	1.95–2.07, m	33.7 (CH_2_)	1.68, td (13.5, 3.5) 1.86–1.96, m
13	27.8 (CH_2_)	1.97–2.07, m	31.0 (CH_2_)	1.49–1.60, m 1.88, td (11.7, 4.3)
14	137.0 (C)	-	80.4 (C)	-
15	127.0 (C)	-	150.5 (C)	-
16	32.8 (CH_2_)	1.90, t (6.2)	34.0 (CH_2_)	1.97, td (13.0, 5.7) 2.32, brd (13.0)
17	19.5 (CH_2_)	1.54–1.59, m	22.7 (CH_2_)	1.43–1.65, m
18	39.9 (CH_2_)	1.39–1.43, m	38.0 (CH_2_)	1.37, brd (13.7) 1.63, td (13.7, 4.5)
19	35.0 (C)	-	39.7 (C)	-
20	28.6 (CH_3_)	0.99, s	24.1 (CH_3_)	0.96, s
21	28.6 (CH_3_)	0.99, s	22.1 (CH_3_)	0.88, s
22	19.8 (CH_3_)	1.59, s	108.3 (CH_2_)	4.83, brs 4.91, brs
23	16.0 (CH_3_)	1.61, s	16.3 (CH_3_)	1.60, s
24	194.2 (CH)	9.43, s	60.3 (CH_2_)	4.10, d (12.1) 4.13, d (12.1)
25	103.3 (CH)	5.84, s	58.6 (CH_2_)	4.44, d (16.9) 4.52, d (16.9)
OMe-25	57.9 (CH_3_)	3.65, s		

Assignments are based on COSY, HSQC (Figure S15) or HMQC (Figure S19), and HMBC experiments.

**Table 3:** ^1^H (600 MHz) and ^13^C (150 MHz) NMR data (CDCl_3_) of compounds **5** and **6**.

Position	5			6		
	δ_C_ (Type)		δ_H_, Mult. (*J* in Hz)	δ_C_ (Type)		δ_H_, Mult. (*J* in Hz)
2	173.8 (C)		-	173.9 (C)		-
3	117.3 (CH)		5.93, s	117.2 (CH)		5.93, s
4	167.3 (C)		-	167.4 (C)		-
5	73.8 (CH_2_)		4.85, s	73.8 (CH_2_)		4.85, s
1′	36.3 (CH_2_)		2.52, dd (14.8, 8.2) 2.64, dd (14.8, 3.2)	36.5 (CH_2_)		2.52, dd (15.2, 8.2) 2.64, dd (15.2, 3.4)
2′	70.2 (CH)		3.84–3.90, m	70.2 (CH)		3.84–3.89, m
3′	37.7 (CH_2_)		1.49–1.54, m	37.7 (CH_2_)		1.49–1.55, m
4′	25.5 (CH_2_)		1.28–1.38, m 1.39–1.46, m	25.5 (CH_2_)		1.30–1.47, m
	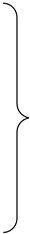	(CH_2_)-5′– (CH_2_)-14′ 30.0, 29.68, 29.64, 29.63, 29.61, 29.57, 29.49, 29.49, 29.48, 27.1 (C-14′)	1.22–1.50, m	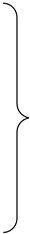	(CH_2_)-5′– (CH_2_)-14′ 29.65, 29.65, 29.65, 29.63, 29.61, 29.61, 29.58, 29.50, 29.50, 29.42	1.22–1.38, m
15′	36.6 (CH_2_)		1.07–1.17, m 1.23–1.37, m	29.3 (CH_2_)		1.22–1.38, m
16′	34.4 (CH)		1.23–1.37, m	31.9 (CH_2_)		1.22–1.31, m
17′	29.42 (CH_2_)		1.22–1.50, m	22.6 (CH_2_)		1.22–1.38, m
18′	11.3 (CH_3_)		0.85, t (7.2)	14.0 (CH_3_)		0.88, t (7.0)
19′	19.2 (CH_3_)		0.84, d (6.0)	-		-

Assignments are based on COSY, HSQC (Figures S23 and S27), and HMBC (Figures S24 and S28) experiments.

**Table 4:** Cytotoxicity of compounds **3**, **5**, **7**–**20** and **23** (IC_50_, μM).

Compound	MOLT-3	HepG2	HeLa	HuCCA-1	A549	H69AR	KB	T47D	MDA-MB-231	MRC-5
**3**	15.84	27.91	20.02	24.72	41.12	- ^a^	- ^a^	- ^a^	40.09	- ^a^
**5**	31.75	107.46	28.79	65.57	57.24	- ^a^	- ^a^	- ^a^	89.26	- ^a^
**7**	40.11	I	87.95	I	I	- ^a^	- ^a^	- ^a^	I	- ^a^
**8**	1.77	53.49	8.42	11.02	16.77	- ^a^	- ^a^	- ^a^	39.53	13.00
**9**	1.30	27.91	1.51	8.60	12.00	- ^a^	- ^a^	- ^a^	18.60	10.84
**10**	35.45	I	78.71	I	I	- ^a^	- ^a^	- ^a^	I	- ^a^
**11**	17.38	I	87.04	84.52	I	- ^a^	- ^a^	- ^a^	I	- ^a^
**12**	37.81	I	79.20	119.35	I	- ^a^	- ^a^	- ^a^	I	- ^a^
**13**	34.10	88.73	59.30	94.65	114.30	- ^a^	- ^a^	- ^a^	92.04	- ^a^
**14**	6.68	101.75	21.80	47.38	58.13	- ^a^	- ^a^	- ^a^	84.13	17.48
**15**	- ^a^	- ^a^	63.08	49.07	- ^a^	51.40	58.41	28.04	14.02	- ^a^
**16**	124.32	I	77.54	I	I	- ^a^	- ^a^	- ^a^	I	- ^a^
**17**	- ^a^	- ^a^	63.08	60.75	- ^a^	107.48	63.08	42.06	60.75	- ^a^
**18**	- ^a^	- ^a^	23.32	41.45	- ^a^	56.99	6.99	5.18	5.96	- ^a^
**19**	13.83	75.45	65.32	60.81	60.81	- ^a^	- ^a^	- ^a^	- ^a^	- ^a^
**20**	7.64	27.36	- ^a^	87.06	73.38	- ^a^	- ^a^	- ^a^	- ^a^	70.97
**23**	77.98	I	I	I	I	- ^a^	- ^a^	- ^a^	- ^a^	- ^a^
Doxorubicin	- ^a^	0.36 ± 0.05	0.19 ± 0.00	0.40 ± 0.03	0.35 ± 0.02		1.64 ± 0.15		1.64 ± 0.17	3.16 ± 0.10
Etoposide	0.04 ± 0.01	- ^a^	- ^a^	- ^a^	- ^a^		- ^a^		- ^a^	- ^a^

I = inactive at IC_50_ > 50 µg/mL; ^a^ not determined; etoposide and doxorubicin were used as the reference compounds.

## Supplementary Materials

The following are available online at http://www.mdpi.com/1660-3397/16/12/474/s1, Figure S1. ^1^H NMR spectrum (600 MHz) of compound **1** in C_6_D_6_, Figure S2.^ 13^C NMR spectrum (150 MHz) of compound **1** in C_6_D_6_, Figure S3. HSQC spectrum of compound **1** in C_6_D_6_, Figure S4. HMBC spectrum of compound **1** in C_6_D_6_, Figure S5. ^1^H NMR spectrum (600 MHz) of compound **2** in CDCl_3_, Figure S6. ^1^H NMR spectrum (600 MHz) of compound **2** in C_6_D_6_, Figure S7. ^13^C NMR spectrum (150 MHz) of compound **2** in CDCl_3_, Figure S8. ^13^C NMR spectrum (150 MHz) of compound **2** in C_6_D_6_, Figure S9. HSQC spectrum of compound **2** in CDCl_3_, Figure S10. HSQC spectrum of compound **2** in C_6_D_6_, Figure S11. HMBC spectrum of compound **2** in CDCl_3_, Figure S12. HMBC spectrum of compound **2** in C_6_D_6_, Figure S13. ^1^H NMR spectrum (600 MHz) of compound **3** in CDCl_3_, Figure S14. ^13^C NMR spectrum (150 MHz) of compound **3** in CDCl_3_, Figure S15. HSQC spectrum of compound **3** in CDCl_3_, Figure S16. HMBC spectrum of compound **3** in CDCl_3_, Figure S17. ^1^H NMR spectrum (600 MHz) of compound **4** in CDCl_3_, Figure S18. ^13^C NMR spectrum (150 MHz) of compound **4** in CDCl_3_, Figure S19. HMQC spectrum of compound **4** in CDCl_3_, Figure S20. HMBC spectrum of compound **4** in CDCl_3_, Figure S21. ^1^H NMR spectrum (600 MHz) of compound **5** in CDCl_3_, Figure S22. ^13^C NMR spectrum (150 MHz) of compound **5** in CDCl_3_, Figure S23. HSQC spectrum of compound **5** in CDCl_3_, Figure S24. HMBC spectrum of compound **5** in CDCl_3_, Figure S25. ^1^H NMR spectrum (600 MHz) of compound **6** in CDCl_3_, Figure S26. ^13^C NMR spectrum (150 MHz) of compound **6** in CDCl_3_, Figure S27. HSQC spectrum of compound **6** in CDCl_3_, Figure S28. HMBC spectrum of compound **6** in CDCl_3._
