# Supplementary material for: Cytotoxic Sesterterpenes from Thai Marine Sponge Hyrtios erectus
Source: Mar Drugs. 2018 Nov 28;16(12):474. doi: 10.3390/md16120474 (PMC6316393; doi:10.3390/md16120474)
Supplement: Supplementary file 1 [file marinedrugs-16-00474-s001.pdf]

Supplementary Materials

Cytotoxic Sesterterpenes from Thai Marine Sponge *Hyrtios erectus*

Wirongrong Kaweetripob [1], Chulabhorn Mahidol [1,2], Pittaya Tuntiwachwuttikul [3], Somsak Ruchirawat [1,2,4] and Hunsa Prawat [1,*]

1 Chulabhorn Research Institute, Kamphaeng Phet 6 Road, Bangkok 10210, Thailand; Kwirongrong@cri.or.th (W.K.); mahidol_natlab@cri.or.th (C.M.); Somsak@cri.or.th (S.R.)
2 Chulabhorn Graduate Institute, Chemical Biology Program. Chulabhorn Royal Academy, Kamphaeng Phet 6 Rood, Bangkok 10210, Thailand
3 Laboratory of Natural Products Chemistry, Faculty of Science and Technology, Phuket Rajabhat University, Phuket 83000, Thailand; pittaya_tun@yahoo.co.th
4 Center of Excellence on Environmental Health and Toxicology (EHT), CHE, Ministry of Education, Bangkok 10210, Thailand
* Correspondence: hunsa@cri.or.th; Tel.: +66-2-553-8982

List of Supplementary Materials

Figure S1. ^{1}H NMR spectrum (600 MHz) of compound **1** in C_6D_6

Figure S2. ^{13}C NMR spectrum (150 MHz) of compound **1** in C_6D_6

Figure S3. HSQC spectrum of compound **1** in C_6D_6

Figure S4. HMBC spectrum of compound **1** in C_6D_6

Figure S5. ^{1}H NMR spectrum (600 MHz) of compound **2** in $CDCl_3$

Figure S6. ^{1}H NMR spectrum (600 MHz) of compound **2** in C_6D_6

Figure S7. ^{13}C NMR spectrum (150 MHz) of compound **2** in $CDCl_3$

Figure S8. ^{13}C NMR spectrum (150 MHz) of compound **2** in C_6D_6

Figure S9. HSQC spectrum of compound **2** in $CDCl_3$

Figure S10. HSQC spectrum of compound **2** in C_6D_6

Figure S11. HMBC spectrum of compound **2** in $CDCl_3$

Figure S12. HMBC spectrum of compound **2** in C_6D_6

Figure S13. ^{1}H NMR spectrum (600 MHz) of compound **3** in $CDCl_3$

Figure S14. ^{13}C NMR spectrum (150 MHz) of compound **3** in $CDCl_3$

Figure S15. HSQC spectrum of compound **3** in $CDCl_3$

Figure S16. HMBC spectrum of compound **3** in $CDCl_3$

Figure S17. ^{1}H NMR spectrum (600 MHz) of compound **4** in $CDCl_3$

Figure S18. ^{13}C NMR spectrum (150 MHz) of compound **4** in $CDCl_3$

Figure S19. HMQC spectrum of compound **4** in $CDCl_3$

Figure S20. HMBC spectrum of compound **4** in $CDCl_3$

Figure S21. ^{1}H NMR spectrum (600 MHz) of compound **5** in $CDCl_3$

Figure S22. ^{13}C NMR spectrum (150 MHz) of compound **5** in $CDCl_3$

Figure S23. HSQC spectrum of compound **5** in $CDCl_3$

Figure S24. HMBC spectrum of compound **5** in $CDCl_3$

Figure S25. ^{1}H NMR spectrum (600 MHz) of compound **6** in $CDCl_3$

Figure S26. ^{13}C NMR spectrum (150 MHz) of compound **6** in $CDCl_3$

Figure S27. HSQC spectrum of compound **6** in $CDCl_3$

Figure S28. HMBC spectrum of compound **6** in $CDCl_3$

Supplementary Material

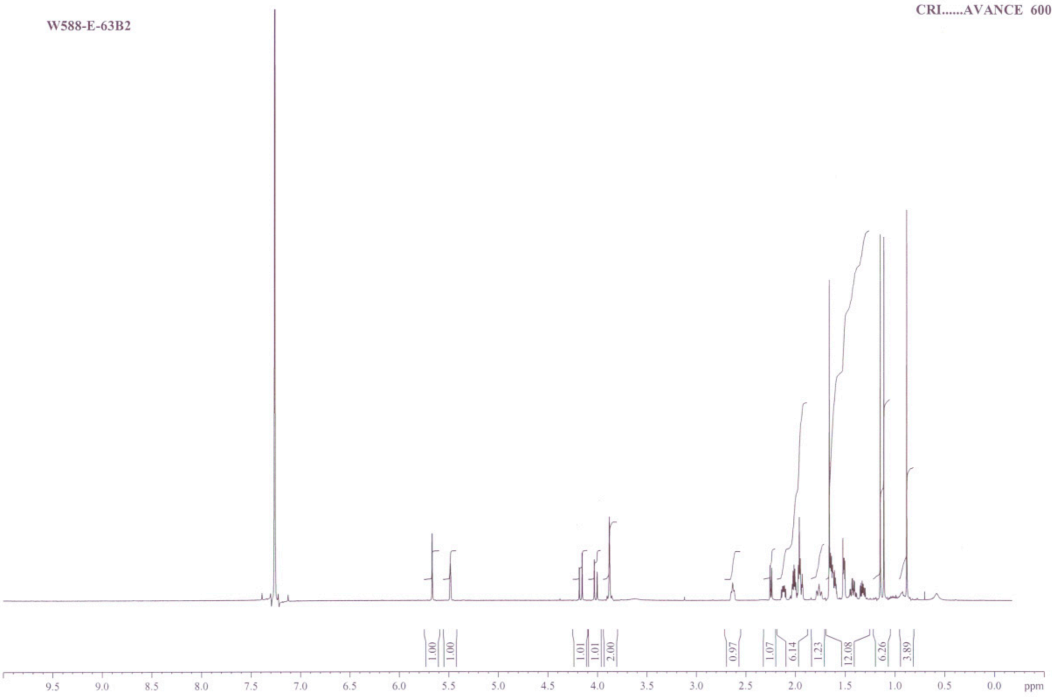

Figure S1. ^{1}H NMR spectrum (600 MHz) of compound **1** in C_6D_6

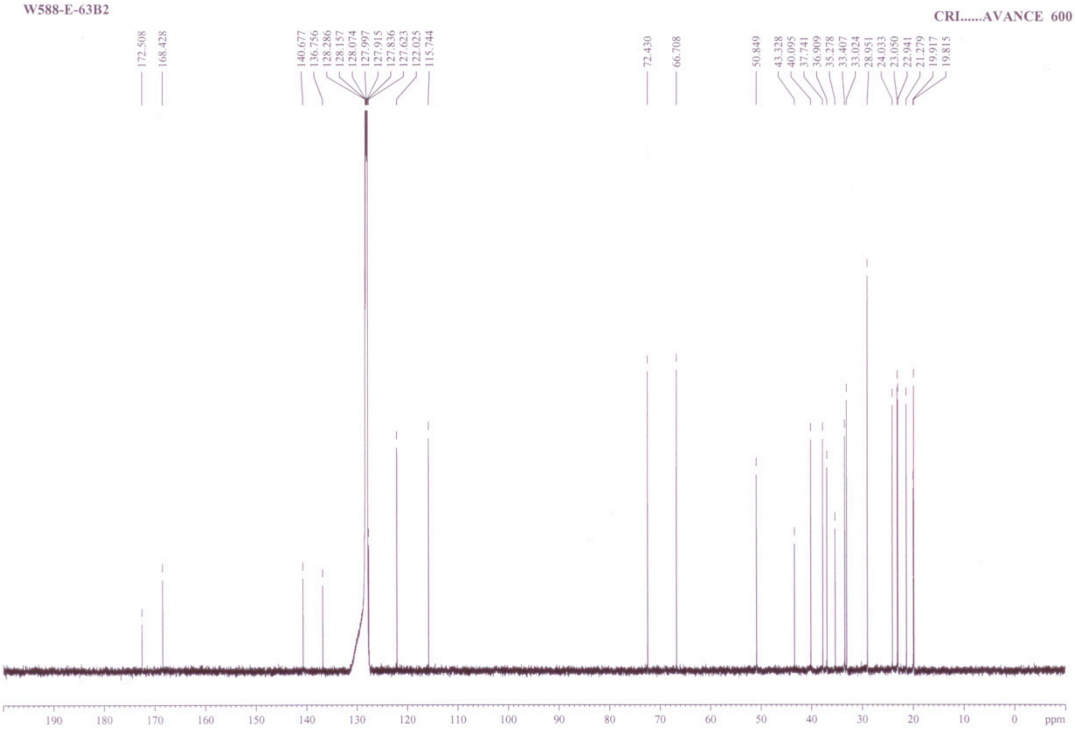

Figure S2. ^{13}C NMR spectrum (150 MHz) of compound **1** in C_6D_6

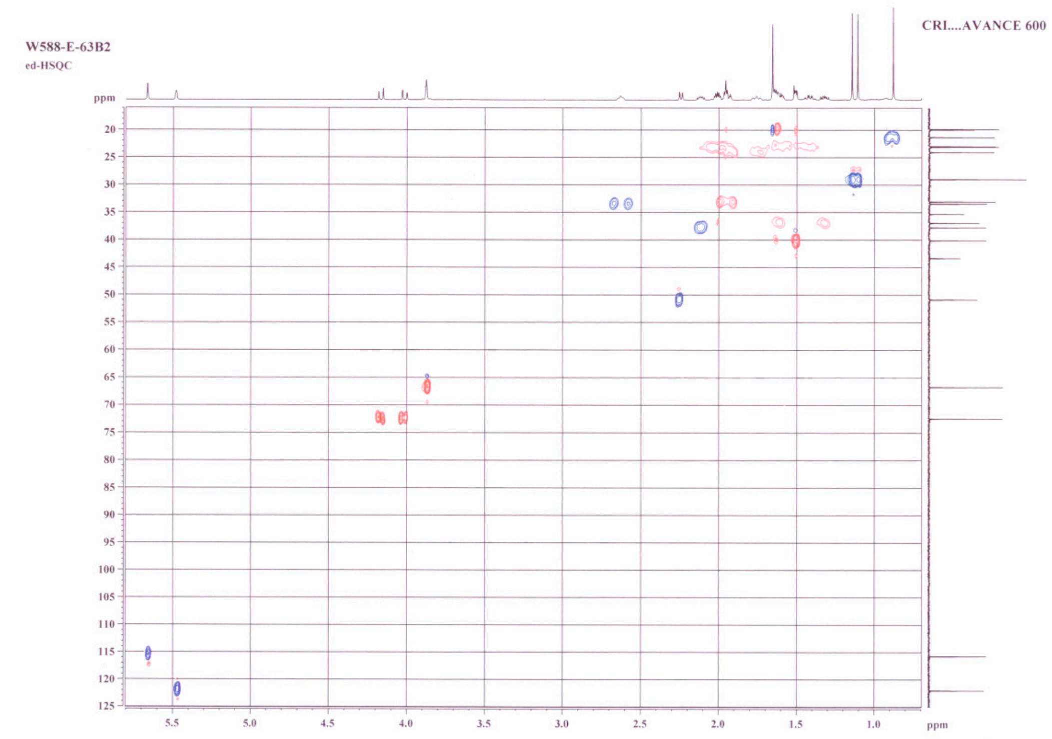

Figure S3. HSQC spectrum of compound **1** in C_6D_6

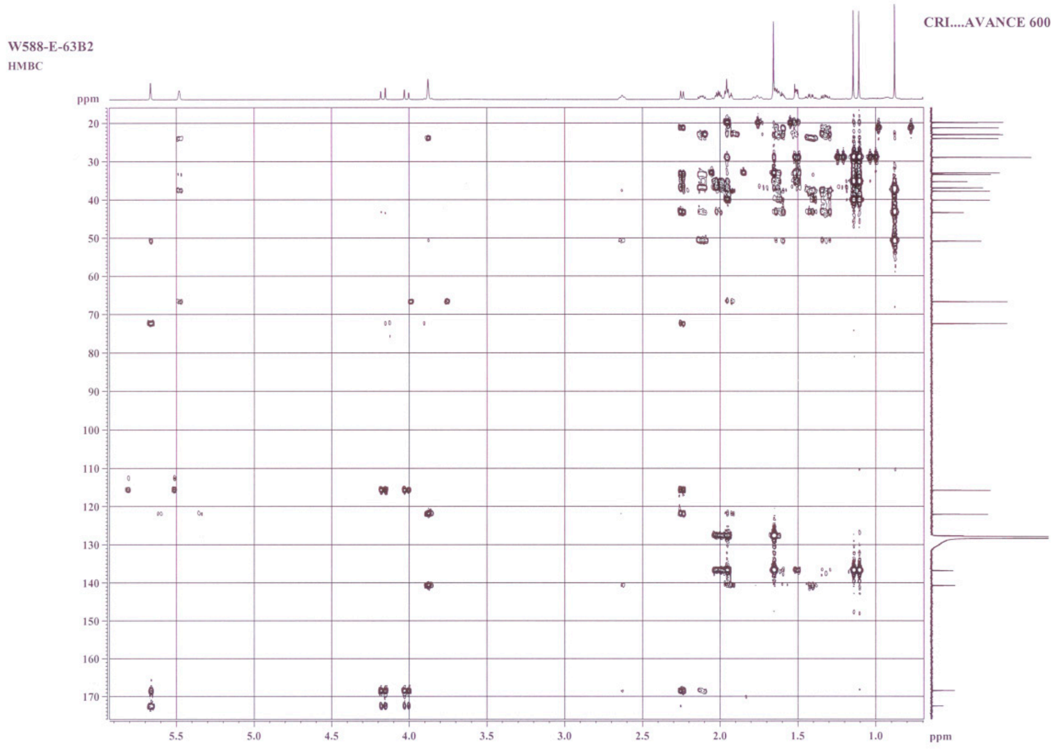

Figure S4. HMBC spectrum of compound **1** in C_6D_6

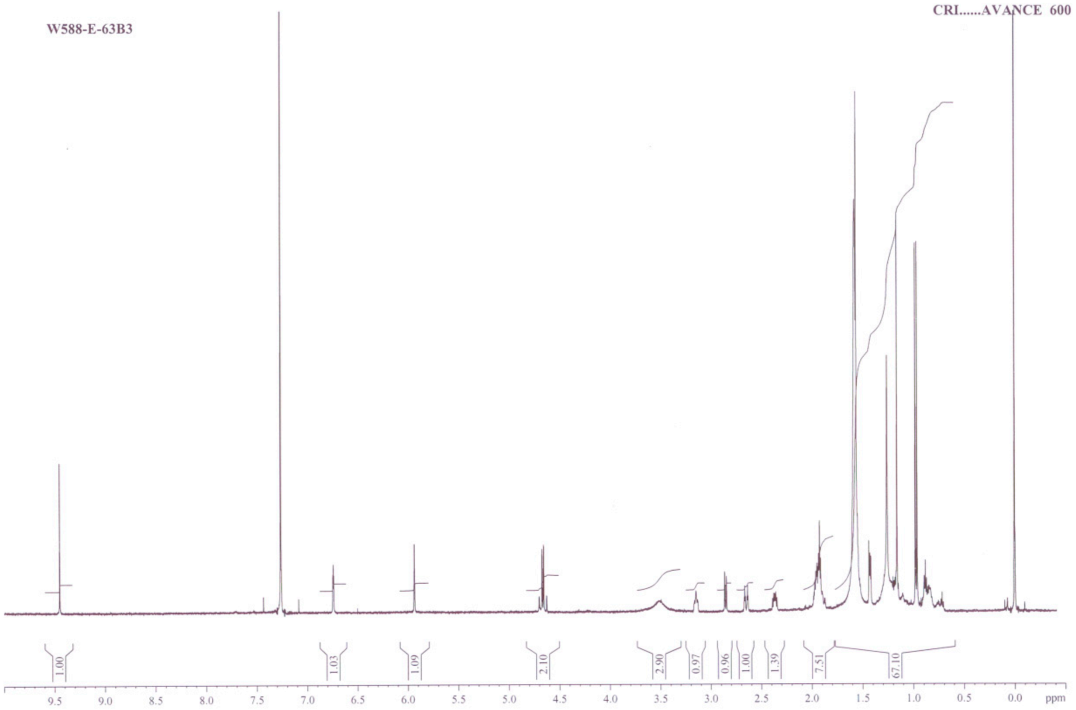

Figure S5. ^{1}H NMR spectrum (600 MHz) of compound **2** in $CDCl_3$

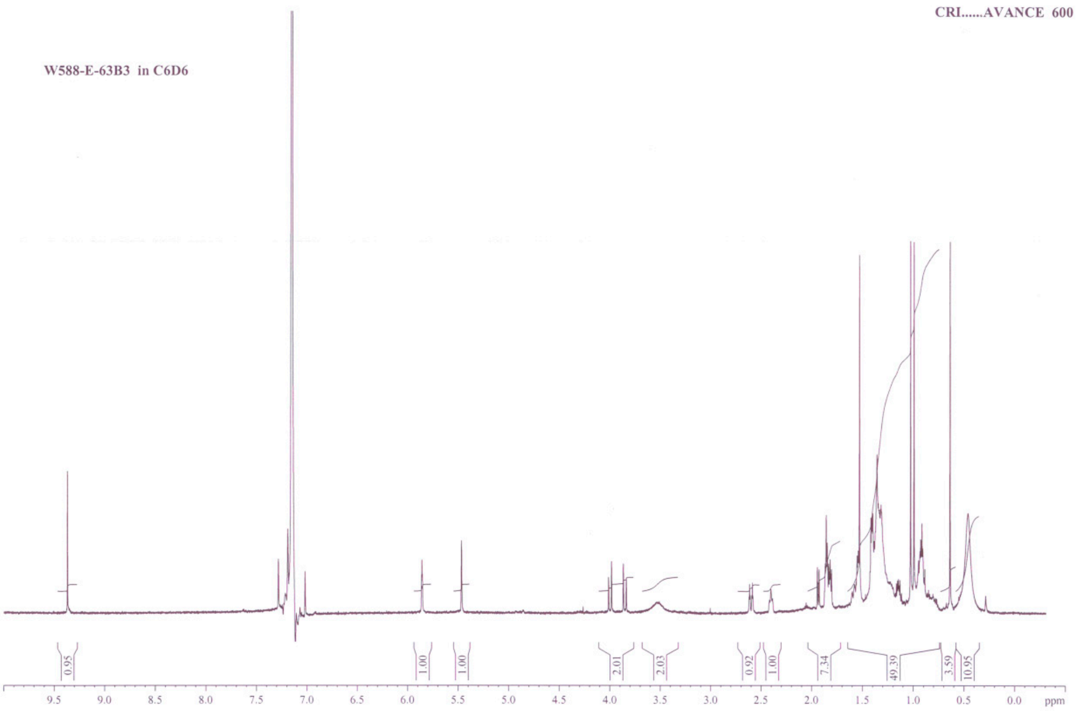

Figure S6. ^{1}H NMR spectrum (600 MHz) of compound **2** in C_6D_6

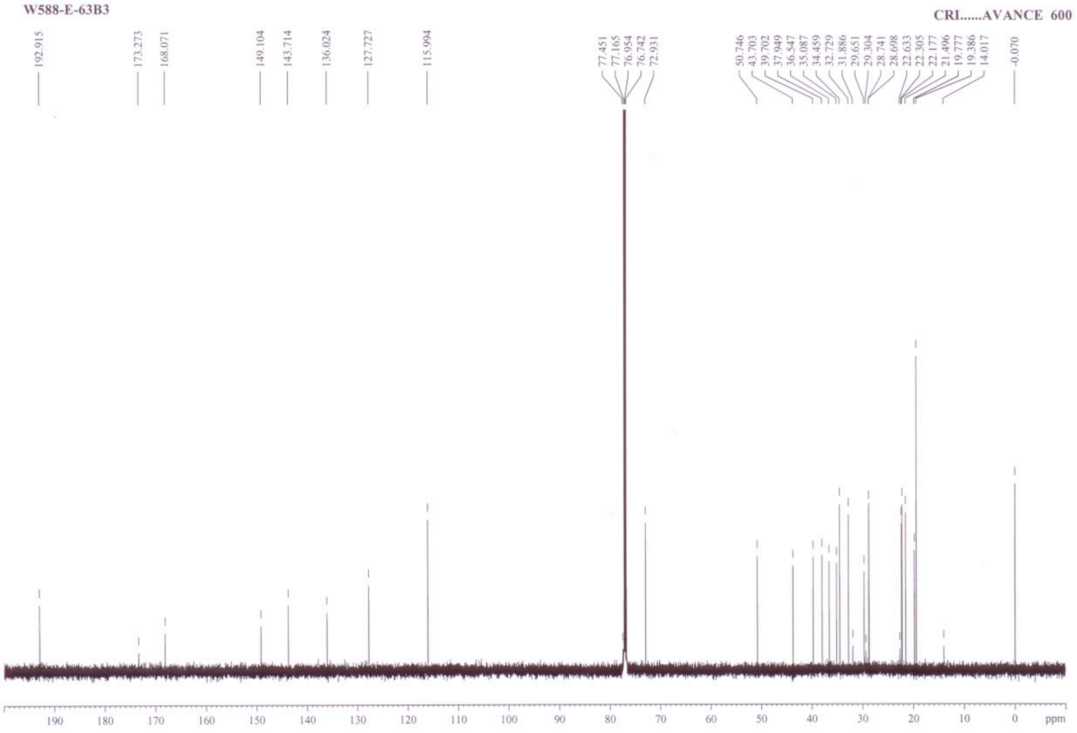

Figure S7. ^{13}C NMR spectrum (150 MHz) of compound **2** in $CDCl_3$

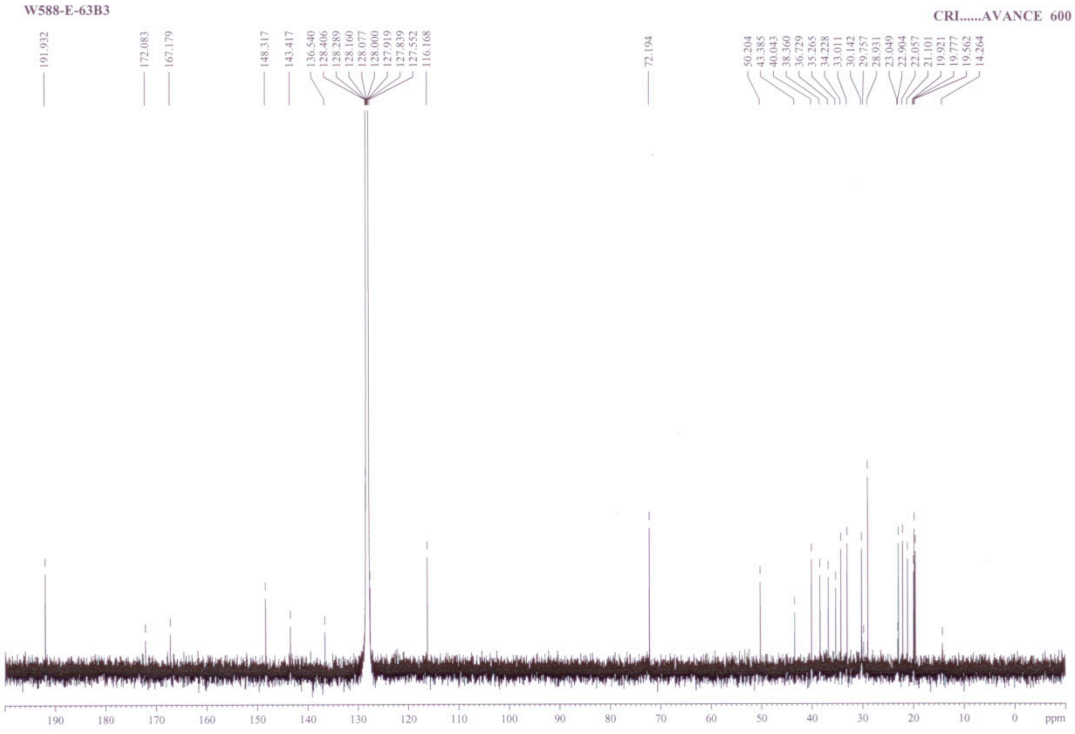

Figure S8. ^{13}C NMR spectrum (150 MHz) of compound **2** in C_6D_6

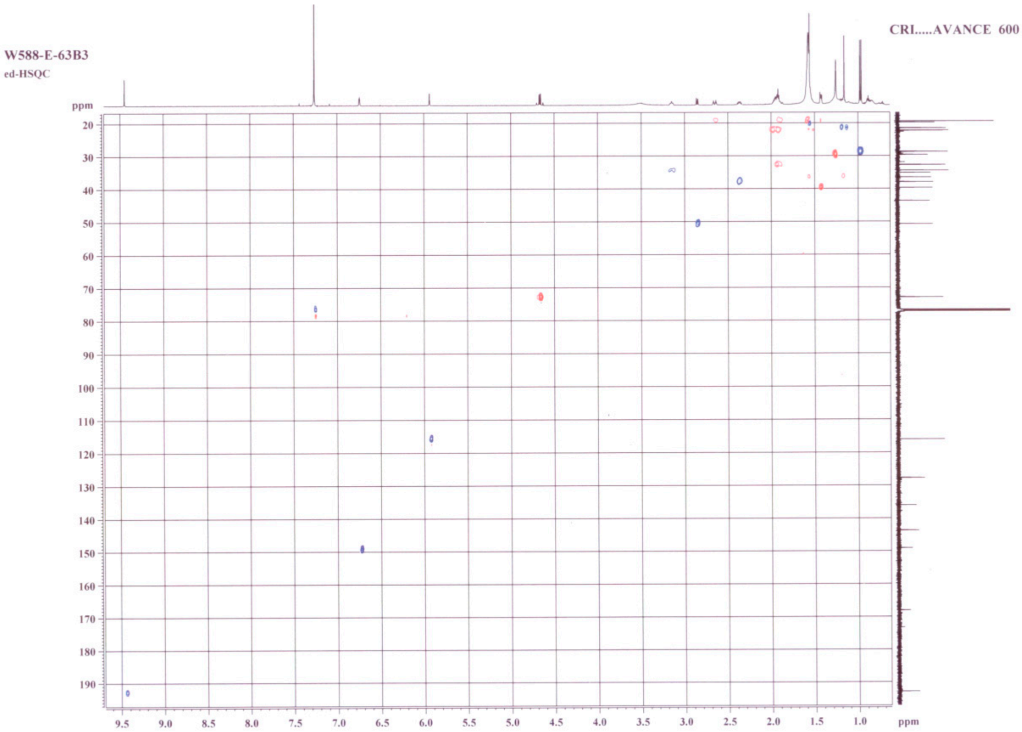

Figure S9. HSQC spectrum of compound **2** in $CDCl_3$

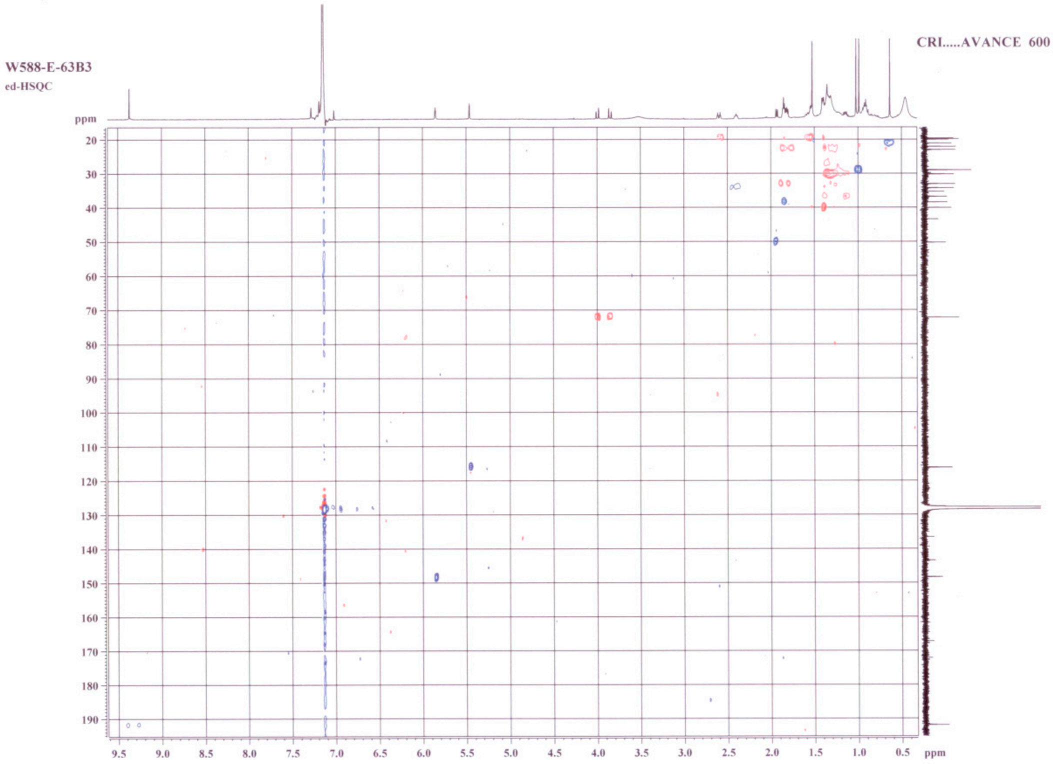

Figure S10. HSQC spectrum of compound **2** in C_6D_6

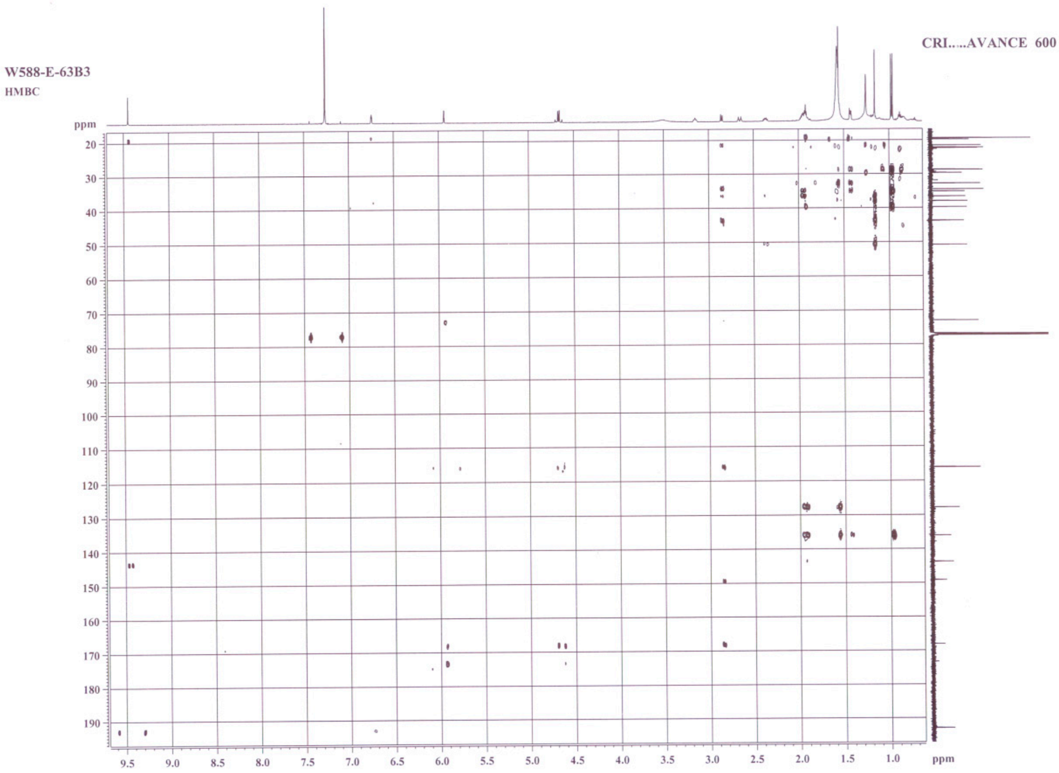

Figure S11. HMBC spectrum of compound **2** in $CDCl_3$

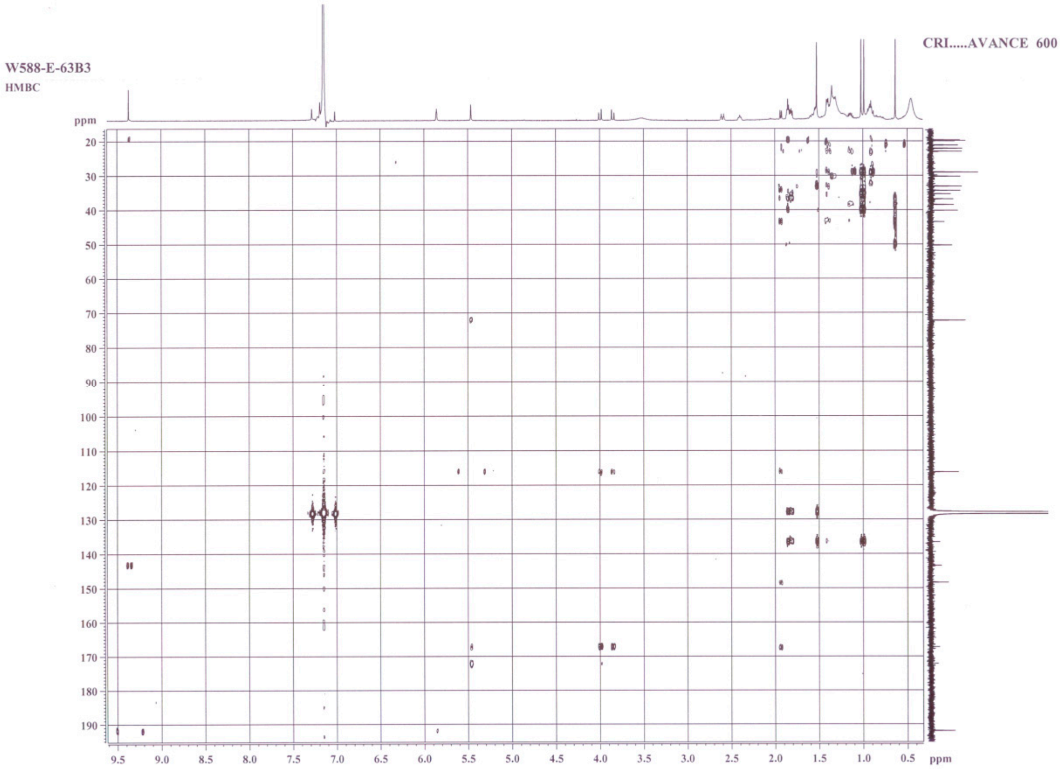

Figure S12. HMBC spectrum of compound **2** in C_6D_6

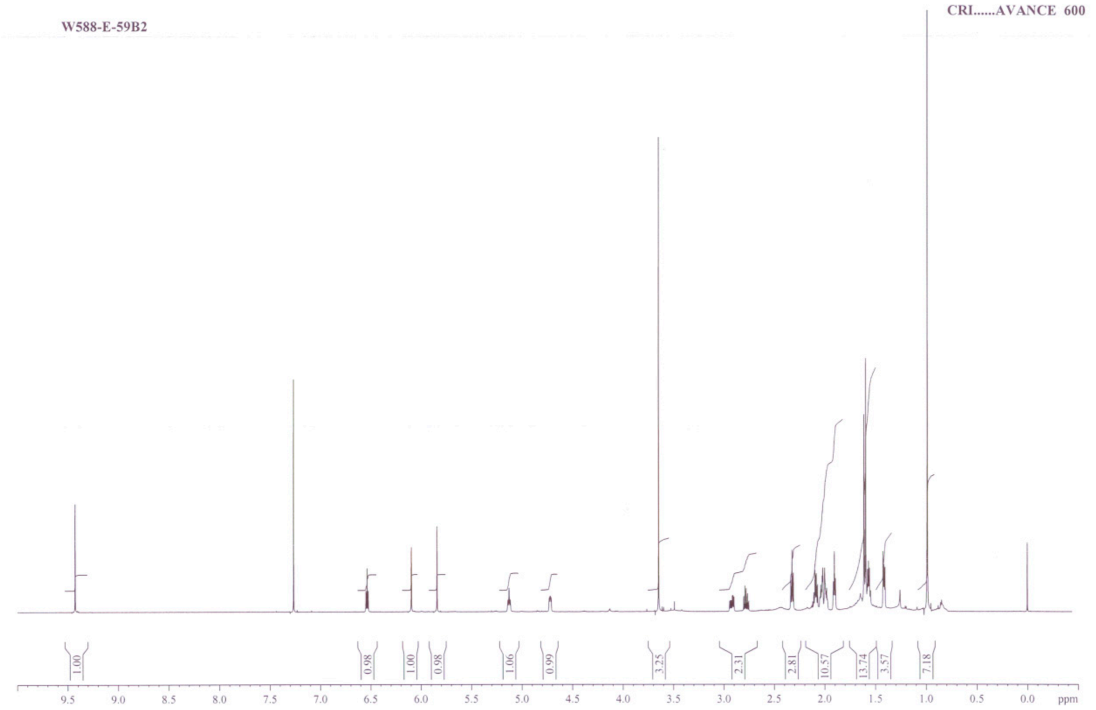

Figure S13. ^{1}H NMR spectrum (600 MHz) of compound **3** in $CDCl_3$

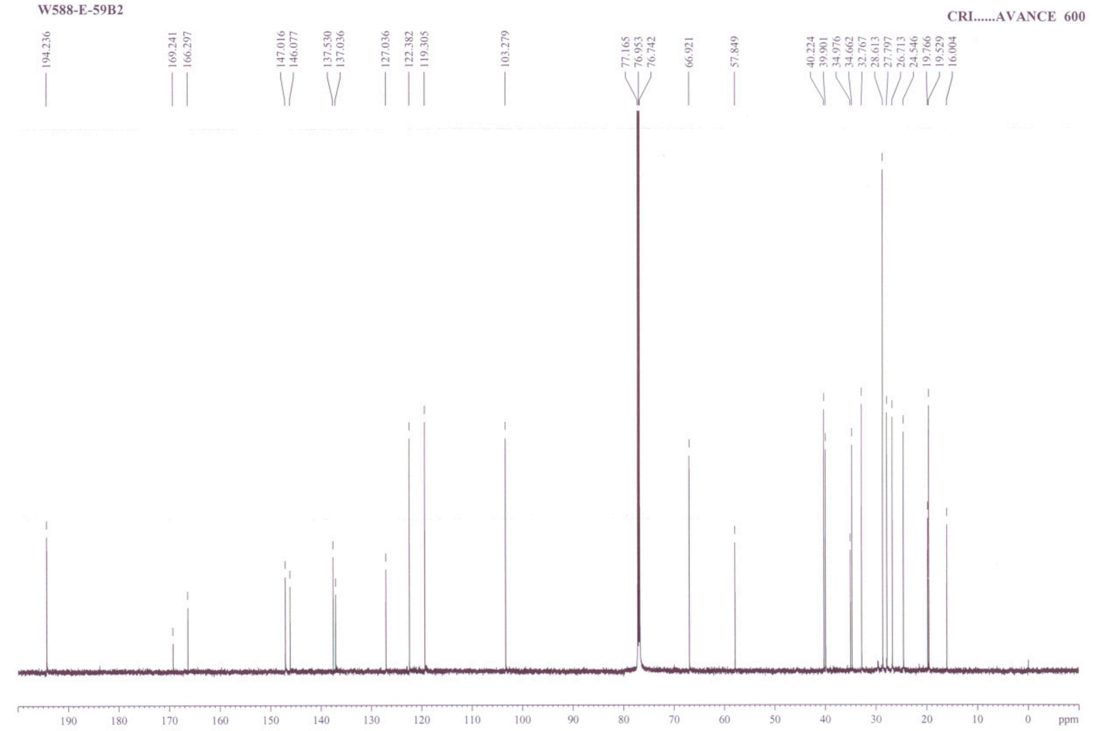

Figure S14. ^{13}C NMR spectrum (150 MHz) of compound **3** in $CDCl_3$

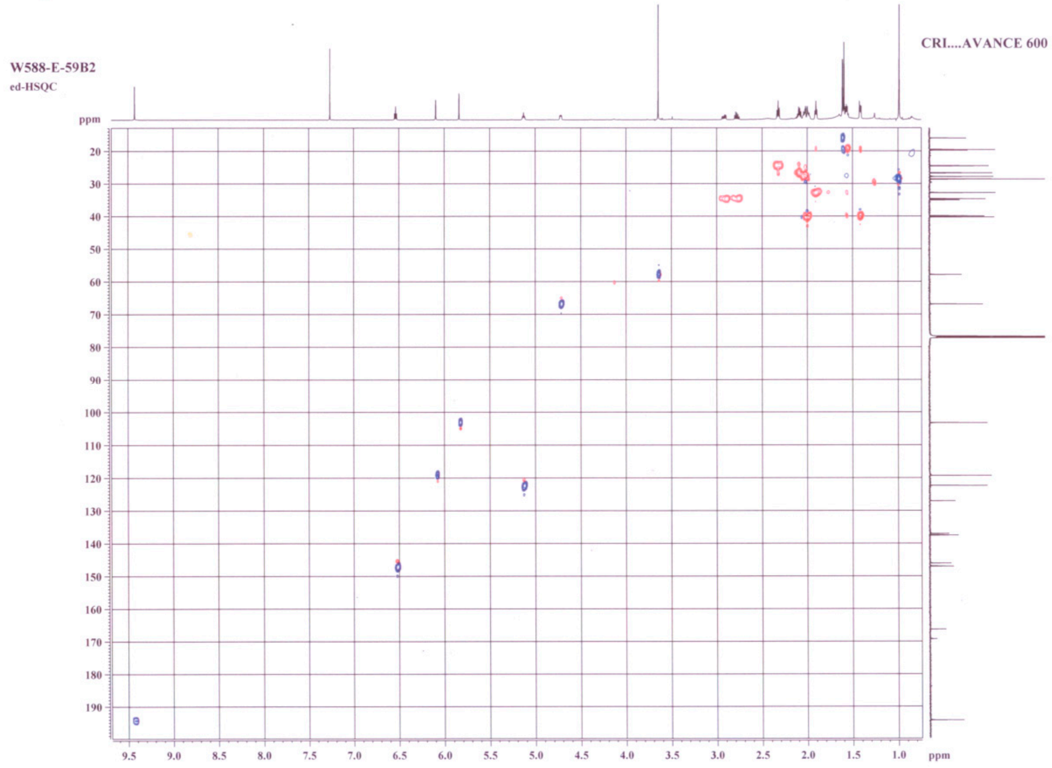

Figure S15. HSQC spectrum of compound **3** in $CDCl_3$

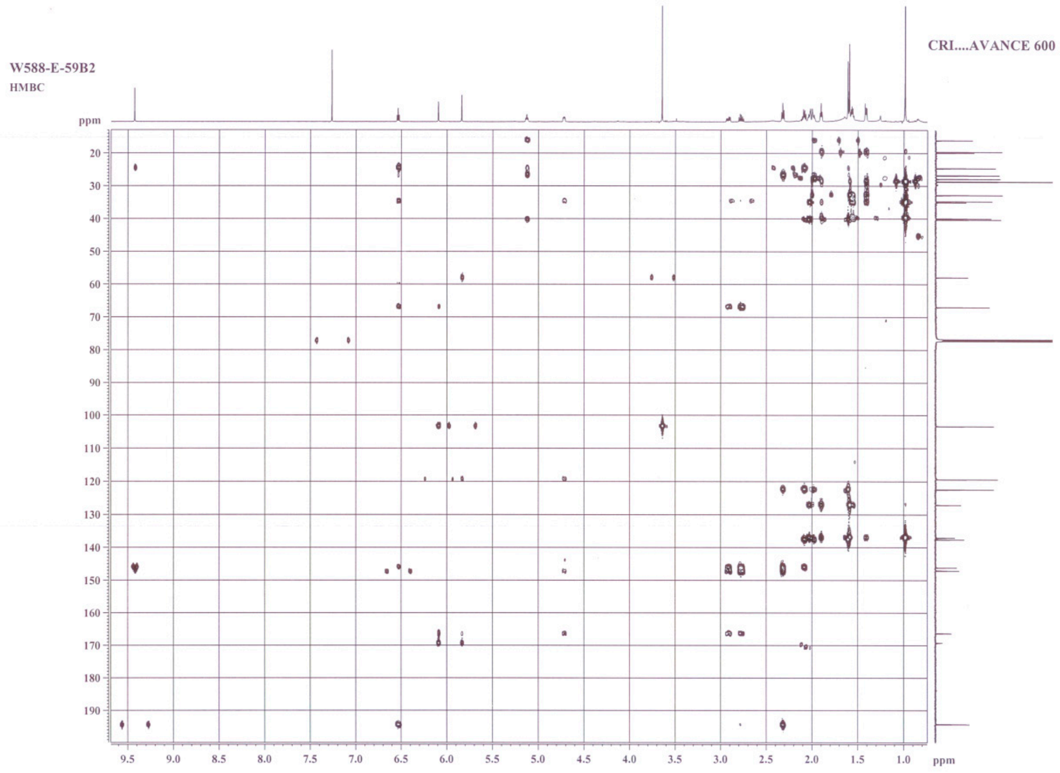

Figure S16. HMBC spectrum of compound **3** in $CDCl_3$

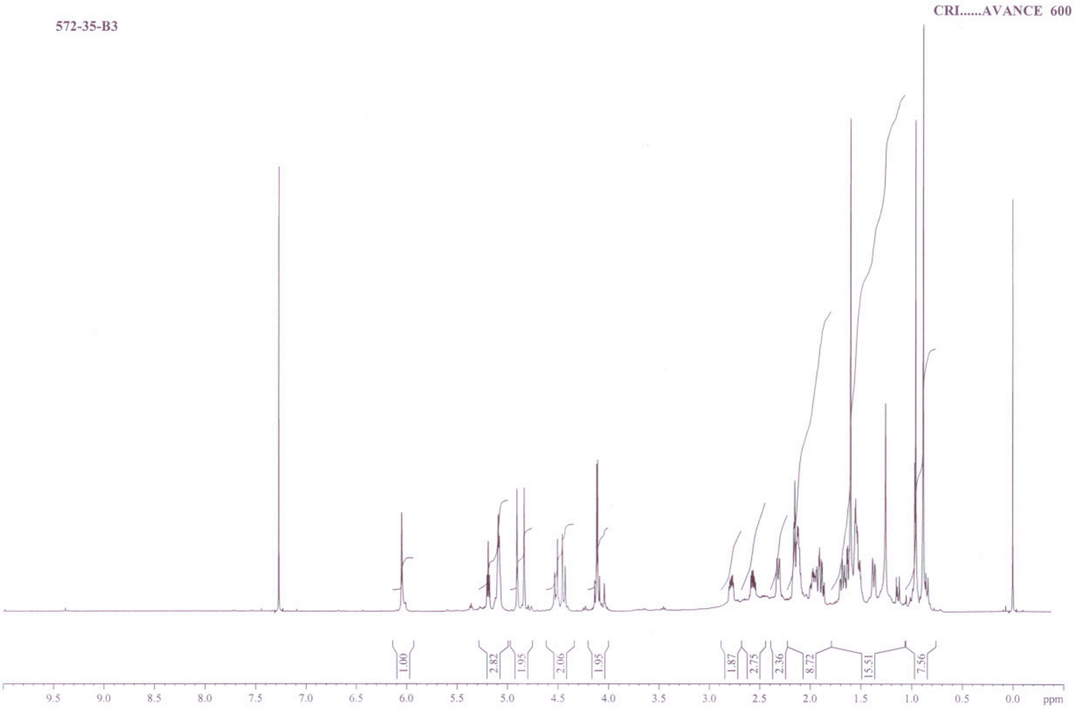

Figure S17. ^{1}H NMR spectrum (600 MHz) of compound **4** in $CDCl_3$

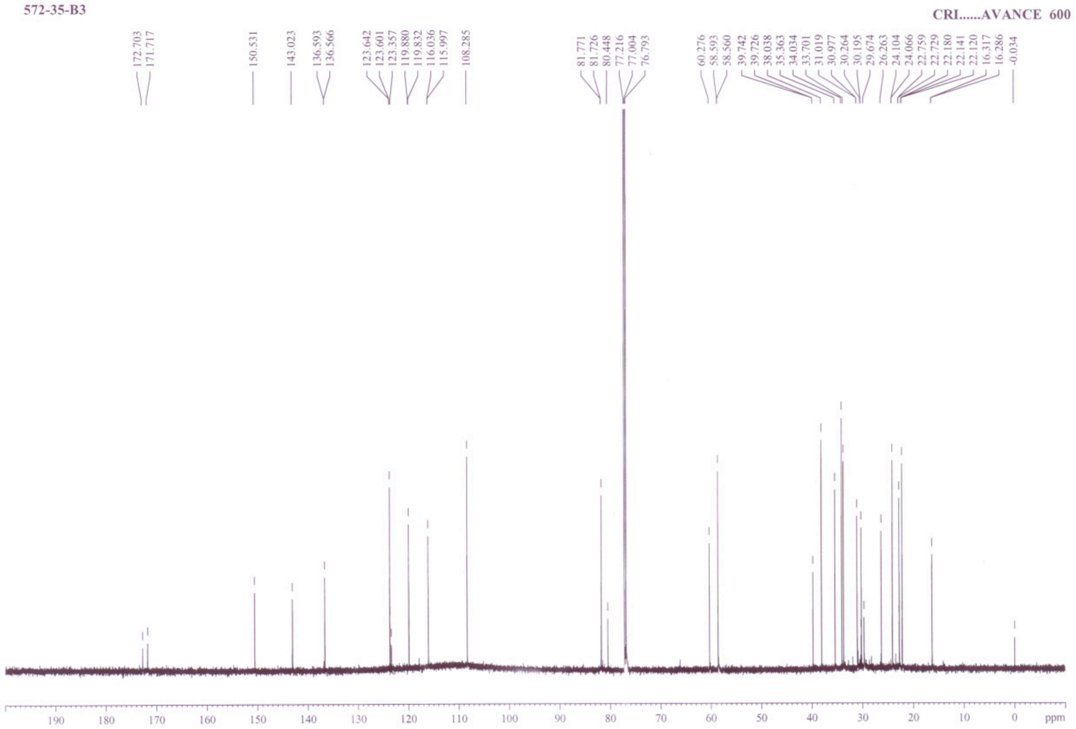

Figure S18. ^{13}C NMR spectrum (150 MHz) of compound **4** in $CDCl_3$

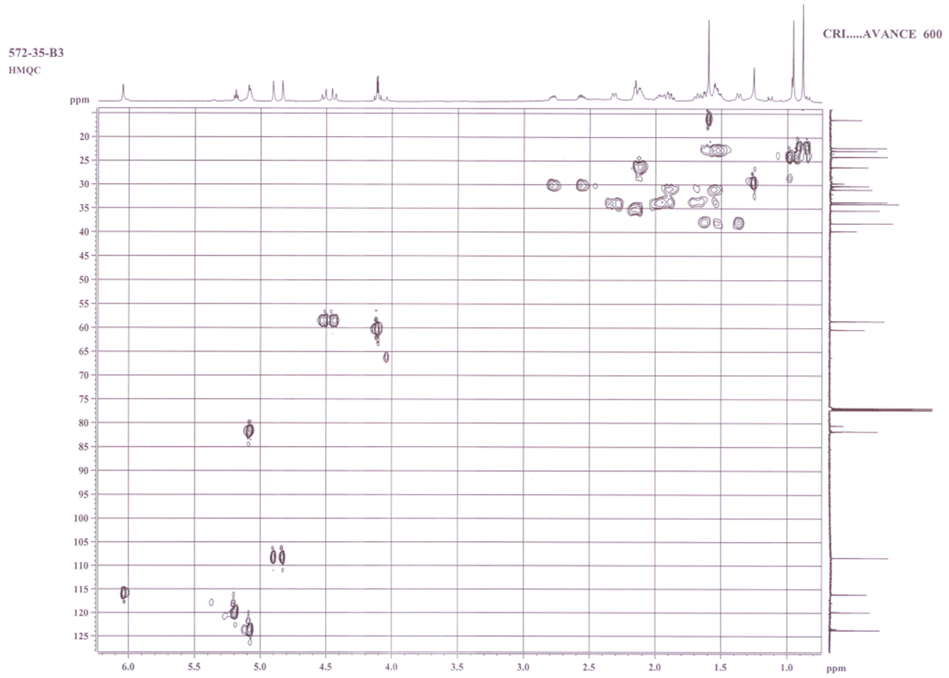

Figure S19. HMQC spectrum of compound **4** in $CDCl_3$

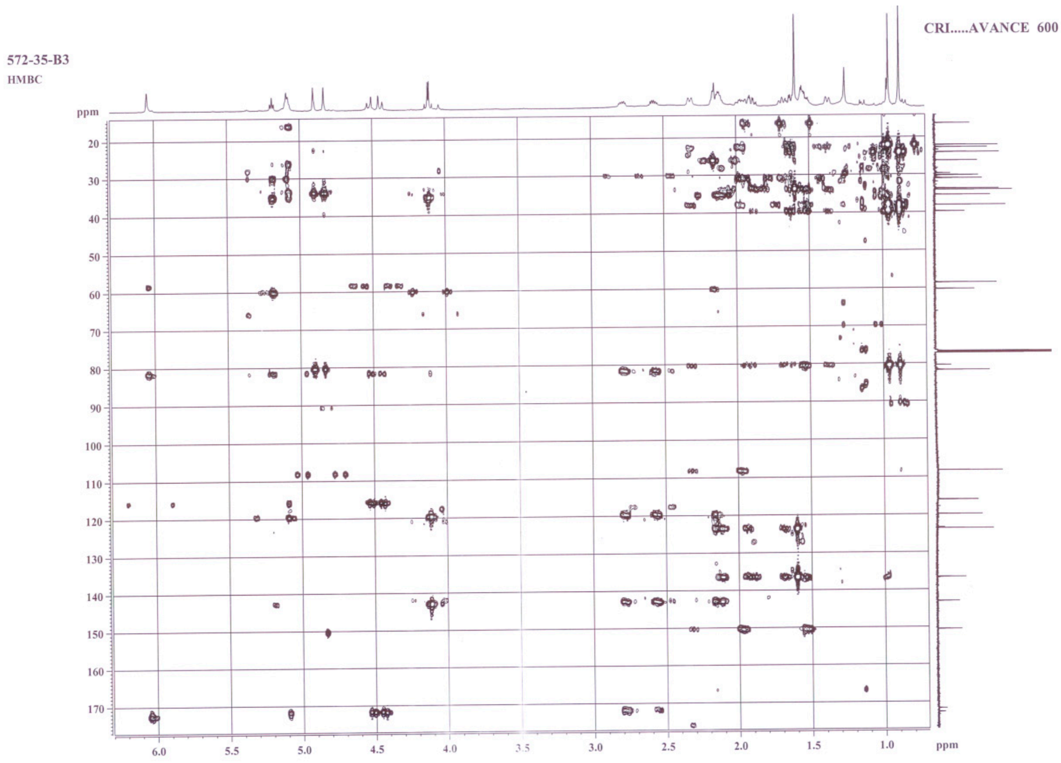

Figure S20. HMBC spectrum of compound **4** in $CDCl_3$

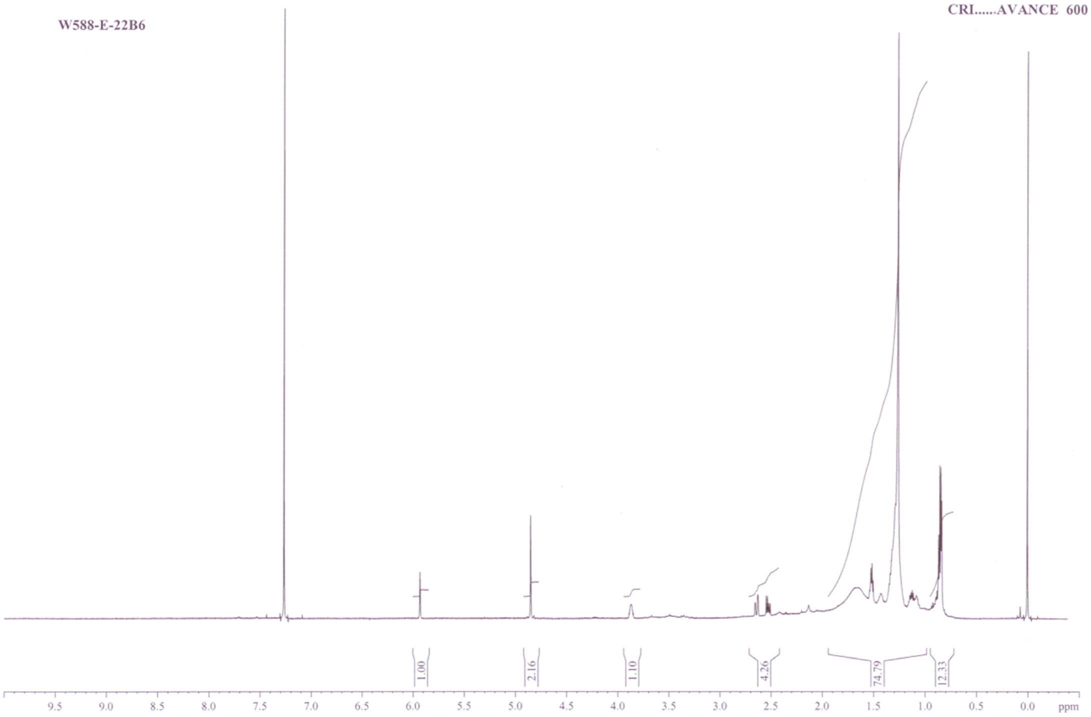

Figure S21. ^{1}H NMR spectrum (600 MHz) of compound **5** in $CDCl_3$

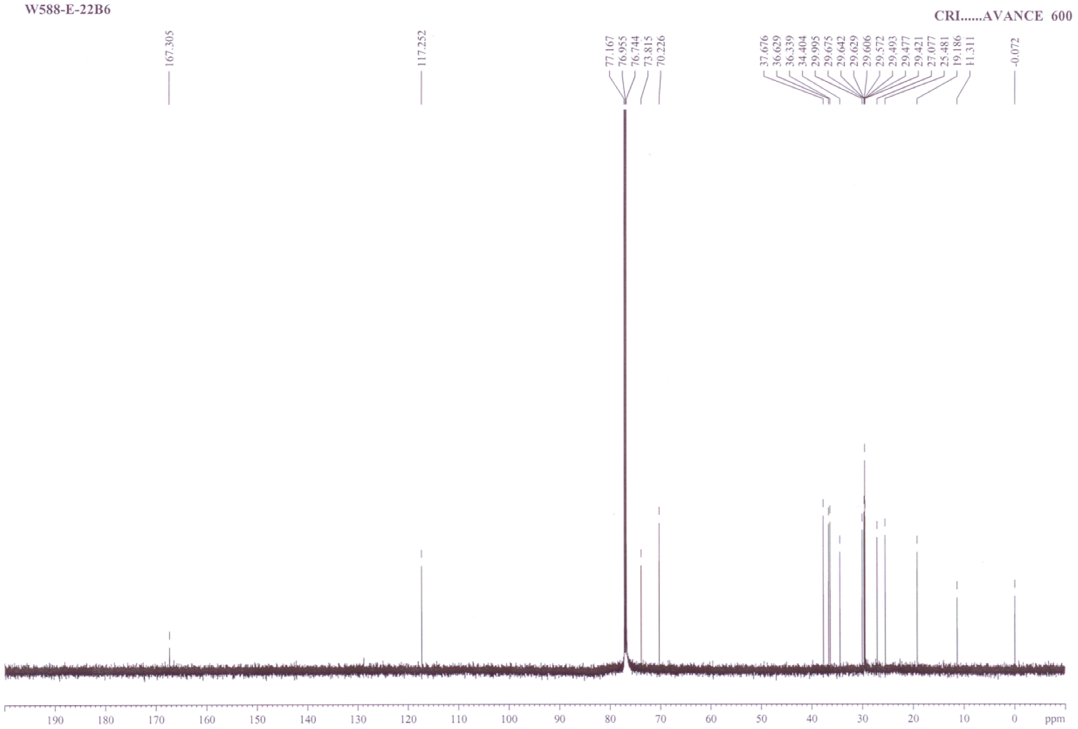

Figure S22. ^{13}C NMR spectrum (150 MHz) of compound **5** in $CDCl_3$

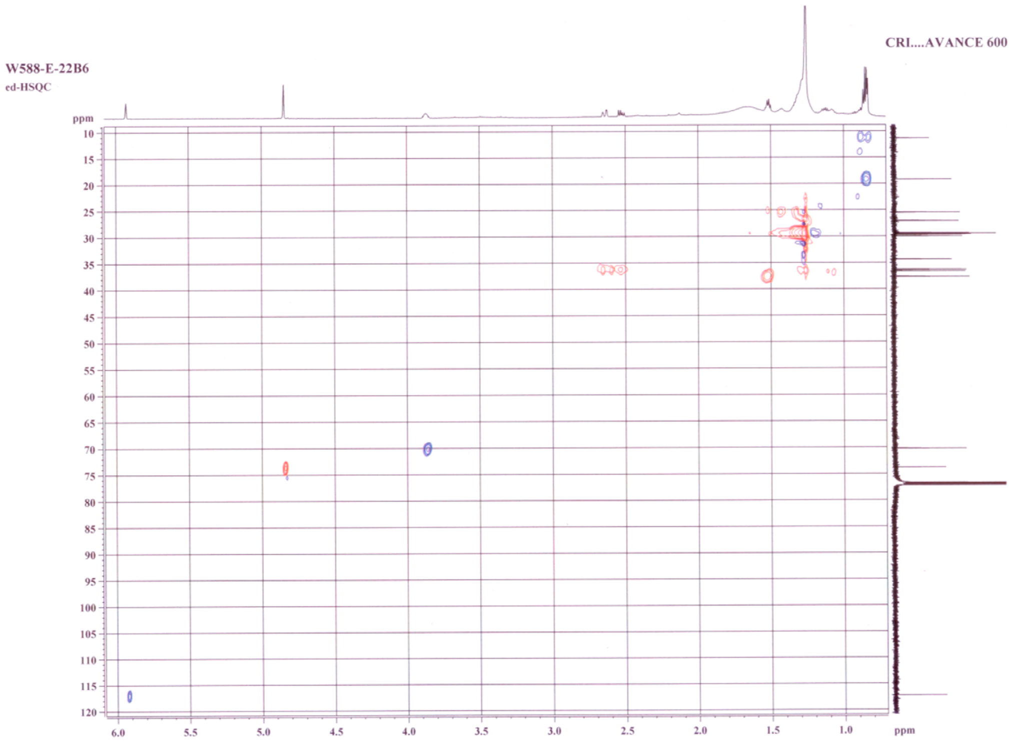

Figure S23. HSQC spectrum of compound **5** in $CDCl_3$

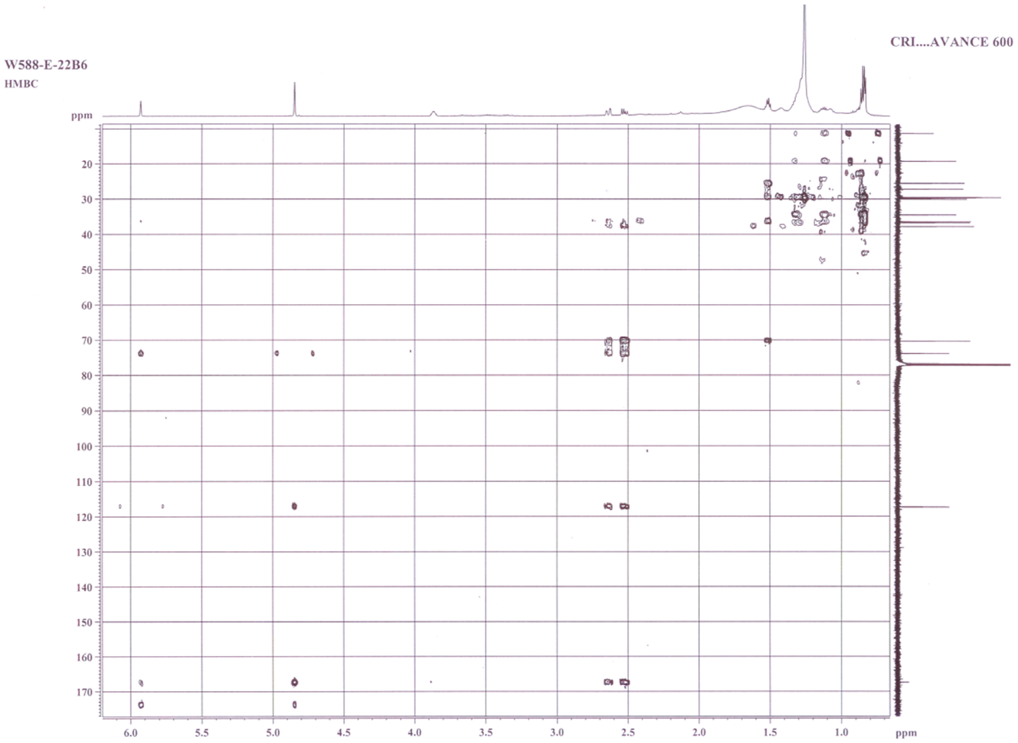

Figure S24. HMBC spectrum of compound **5** in $CDCl_3$

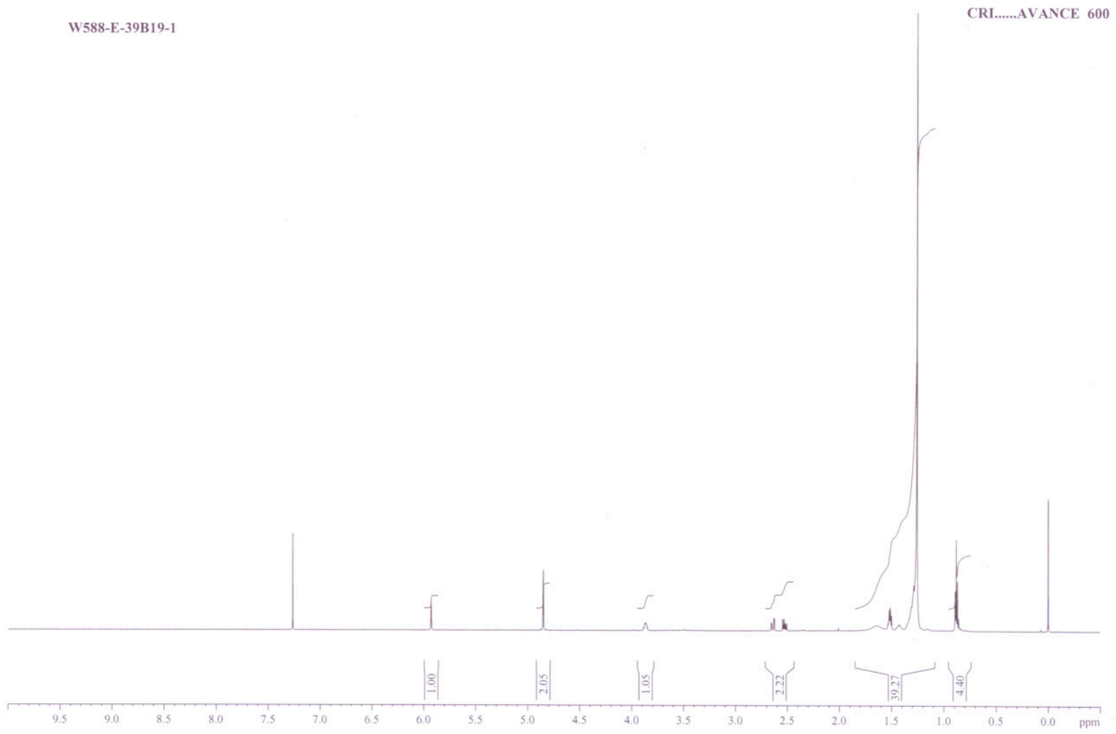

Figure S25. ^{1}H NMR spectrum (600 MHz) of compound **6** in $CDCl_3$

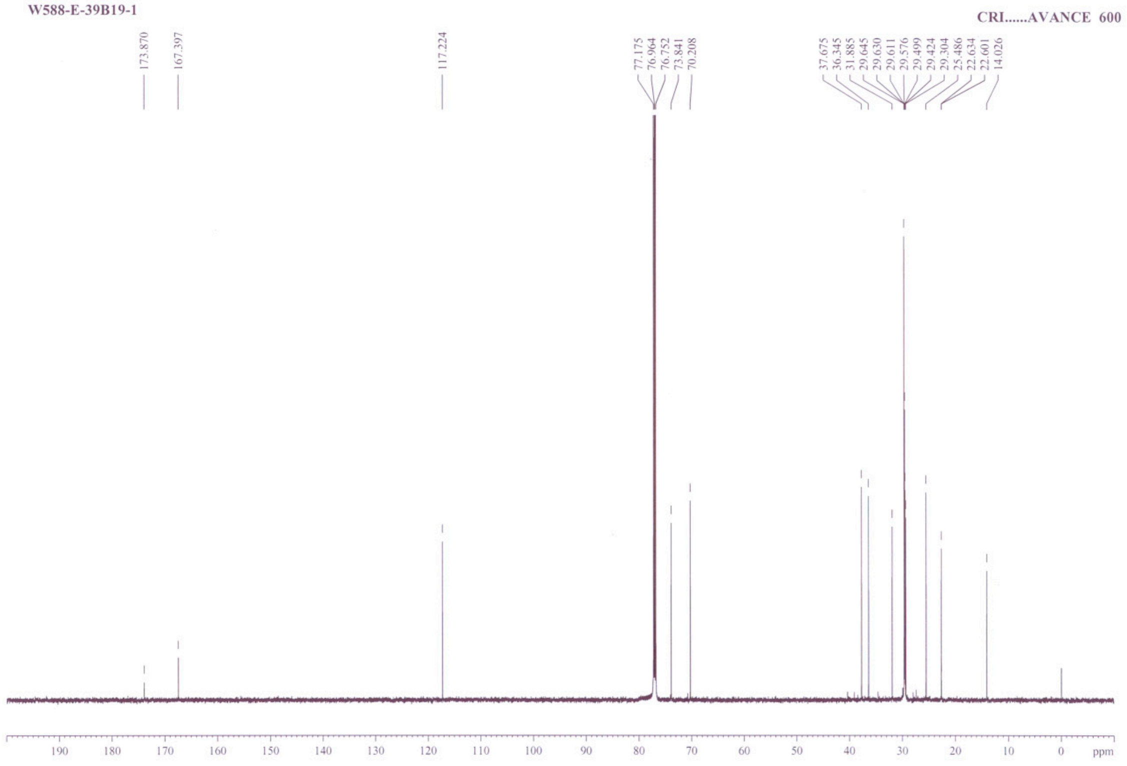

Figure S26. ^{13}C NMR spectrum (150 MHz) of compound **6** in $CDCl_3$

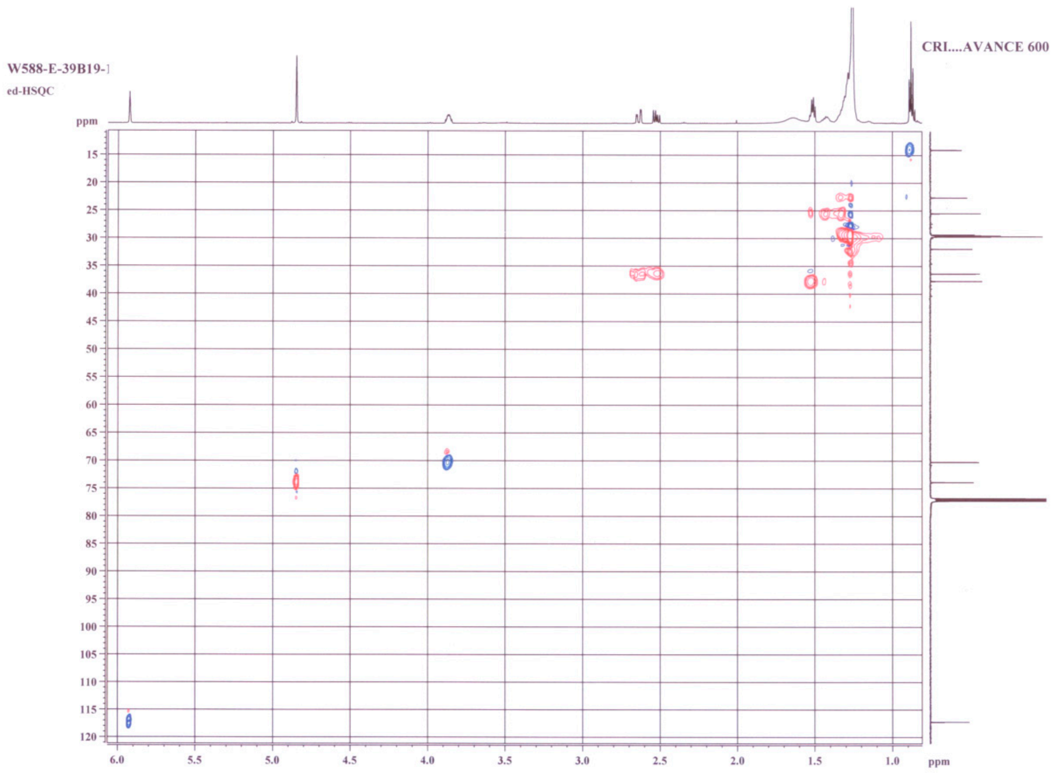

Figure S27. HSQC spectrum of compound **6** in $CDCl_3$

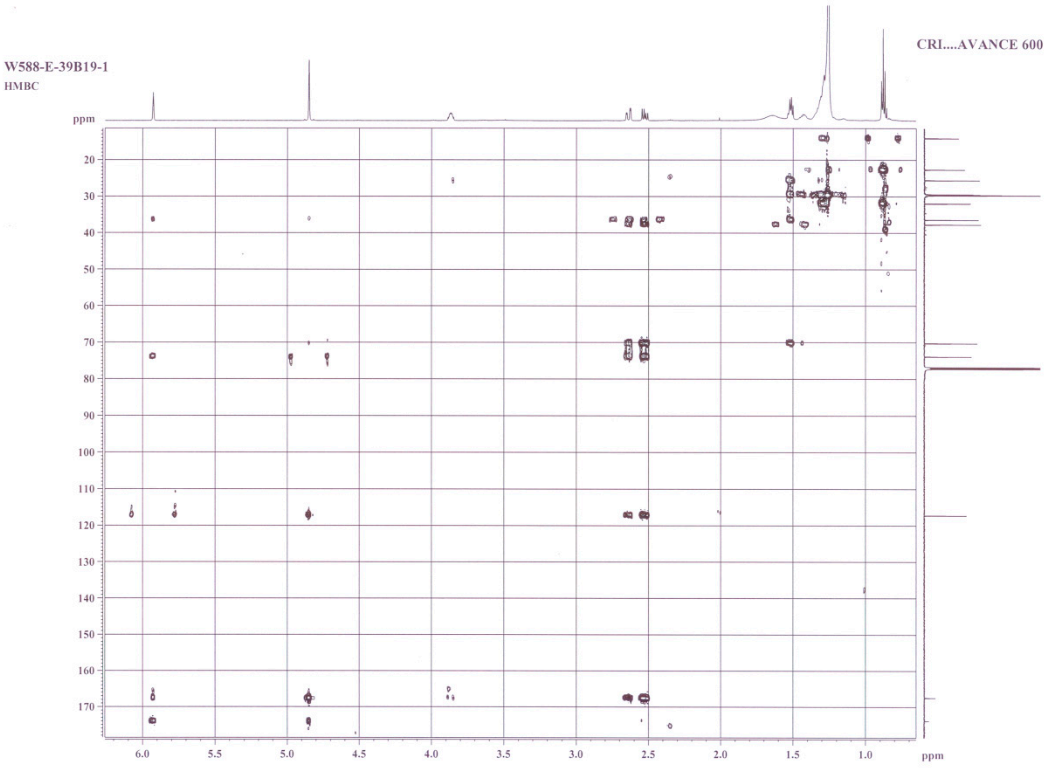

Figure S28. HMBC spectrum of compound **6** in $CDCl_3$
